# Catabolism and Detoxification of 1-Aminoalkylphosphonic Acids: *N*-Acetylation by the *phnO* Gene Product

**DOI:** 10.1371/journal.pone.0046416

**Published:** 2012-10-03

**Authors:** Bjarne Hove-Jensen, Fern R. McSorley, David L. Zechel

**Affiliations:** Department of Chemistry, Queen's University, Kingston, Ontario, Canada; Arizona State University, United States of America

## Abstract

In *Escherichia coli* uptake and catabolism of organophosphonates are governed by the *phnCDEFGHIJKLMNOP* operon. The *phnO* cistron is shown to encode aminoalkylphosphonate *N*-acetyltransferase, which utilizes acetylcoenzyme A as acetyl donor and aminomethylphosphonate, (*S*)- and (*R*)-1-aminoethylphosphonate, 2-aminoethyl- and 3-aminopropylphosphonate as acetyl acceptors. Aminomethylphosphonate, (*S*)-1-aminoethylphosphonate, 2-aminoethyl- and 3-aminopropylphosphonate are used as phosphate source by *E. coli phn^+^* strains. 2-Aminoethyl- or 3-aminopropylphosphonate but not aminomethylphosphonate or (*S*)-1-aminoethylphosphonate is used as phosphate source by *phnO* strains. Neither *phn^+^* nor *phnO* strains can use (*R*)-1-aminoethylphosphonate as phosphate source. Utilization of aminomethylphosphonate or (*S*)-1-aminoethylphosphonate requires the expression of *phnO*. In the absence of *phnO*-expression (*S*)-1-aminoethylphosphonate is bacteriocidal and rescue of *phnO* strains requires the simultaneous addition of d-alanine and phosphate. An intermediate of the carbon-phosphorus lyase pathway, 5′-phospho-α-d-ribosyl 1′-(2-*N*-acetamidoethylphosphonate), a substrate for carbon-phosphorus lyase, was found to accumulate in cultures of a *phnP* mutant strain. The data show that the physiological role of *N*-acetylation by *phnO*-specified aminoalkylphosphonate *N*-acetyltransferase is to detoxify (*S*)-1-aminoethylphosphonate, an analog of d-alanine, and to prepare (*S*)-1-aminoethylphosphonate and aminomethylphosphonate for utilization of the phosphorus-containing moiety.

## Introduction

*Escherichia coli* as well as many other bacterial species are able to use a number of phosphorus-containing compounds as phosphate source. The preferred source is inorganic phosphate ion (P_i_). When P_i_ is low or absent, expression of the operons of the Pho regulon is derepressed and other phosphate sources may be utilized. Thus, phosphate esters and organophosphonates also are P_i_ sources for *E. coli*. Utilization of organophosphonates as P_i_ source by *E. coli* requires the 14-cistron *phnCDEFGHIJKLMNOP* operon, which is a member of the Pho regulon, and which specifies the carbon-phosphorus (C–P) lyase pathway [1], [2]. *E. coli* is capable of utilizing both alkylphosphonates, such as methyl- (MePn), ethyl- (EtPn) and propylphosphonate (PrPn) as well as aminoalkylphosphonates, such as aminomethyl- (AmMePn), 2-aminoethyl- (2AmEtPn) and 3-aminopropylphosphonate (3AmPrPn) as P_i_ source. Utilization of both types of phosphonate is dependent on an ATP-binding cassette transport system (encoded by *phnCDE*) [1], [3], as well as two additional apparent nucleotide-binding domains for organophosphonate transport (encoded by *phnK* and *phnL*) [1], the enzymes C–P lyase (encoded by *phnJ* and presumable also *phnGHI*) [1], [4], PhnM [5], α-d-ribosyl 1,5-*bis*phosphate phosphokinase (encoded by *phnN*) [6] and phosphoribosyl cyclic phosphodiesterase (encoded by *phnP*) [7]. The *phnF* cistron likely encodes a repressor of *phn* operon expression [8]. *E. coli phnO* may specify an enzyme with aminoalkylphosphonate *N*-acetyltransferase activity as evaluated by amino acid sequence similarity with the *phnO* gene product of *Salmonella enterica* serotype Typhimurium [9]. The physiological function of *S. enterica* aminoalkylphosphonate *N*-acetyltransferase is presently unknown as this organism does not contain C–P lyase. In contrast, *S. enterica* contains the enzyme phosphonoacetaldehyde phosphohydrolase, which is encoded by the *phnX* gene [10], and which is responsible for the catabolism of 2AmEtPn.

**Figure 1:**
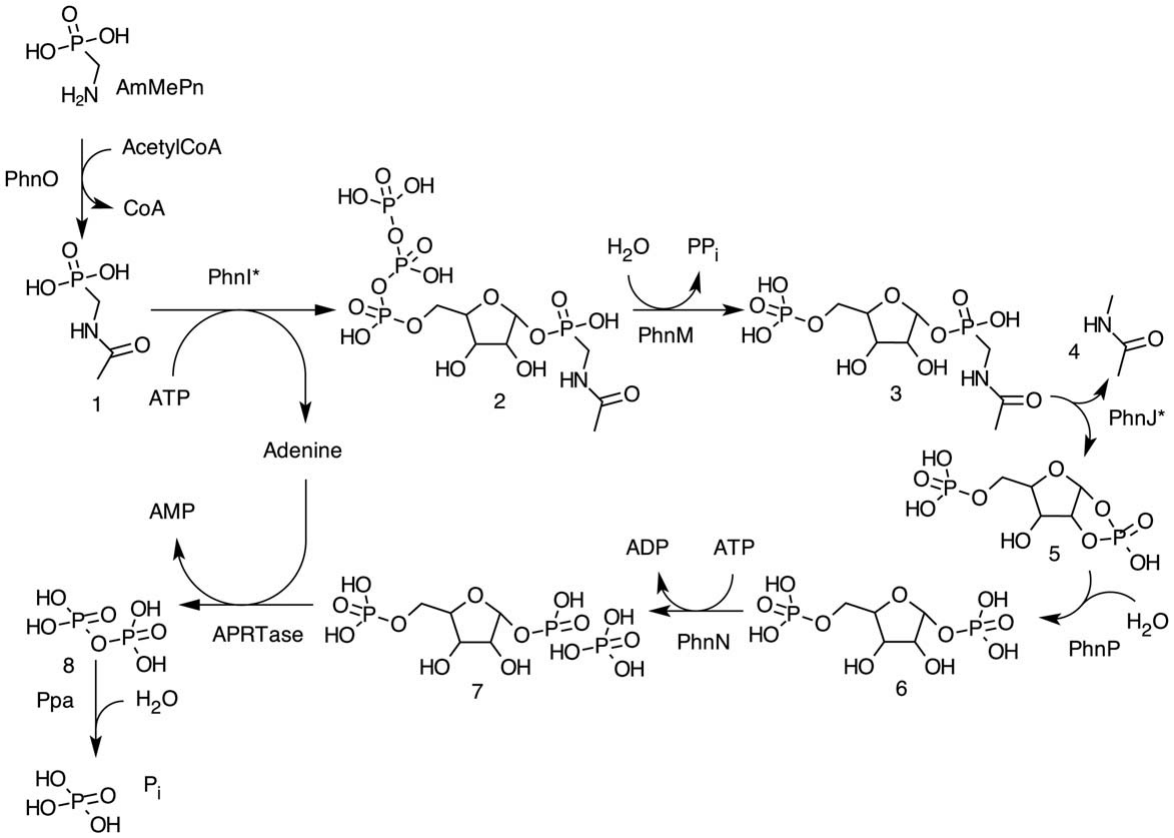
Catabolism of AmMePn. Compounds: 1, NAcAmMePn; 2, 5′-triphospho-α-d-ribosyl 1′-(*N*-acetamidomethylphosphonate); 3, 5′PRib1′NAcAmMePn; 4, *N*-methylacetamide; 5, 5PRib1,2cP; 6, α-d-ribosyl 1,5-*bis*phosphate; 7, PRPP; 8, diphosphate. Reactions are indicated by their enzymes: PhnO, aminoalkylphosphonate *N*-acetyltransferase; PhnI*, an enzyme complex where PhnI plays a crucial catalytic role, and which may involve also PhnG, PhnH, PhnJ, PhnK and/or PhnL; PhnM, 5′-triphospho-α-d-ribosyl 1′-phosphonate diphosphohydrolase; PhnJ*, *S*-adenosylmethionine dependent carbon-phosphorus lyase. PhnJ* may constitute a protein complex containing also PhnG, PhnH, PhnI, PhnK and/or PhnL. PhnI* and PhnJ* may be the same protein complex; PhnP, *phnP* specified phosphoribosyl cyclic phosphodiesterase; PhnN, *phnN* specified ribosyl*bis*phosphate phosphokinase; APRTase, *apt* specified adenine phosphoribosyltransferase; Ppa, *ppa* specified inorganic diphosphate hydrolase. The enzymes of the latter two reactions are not specified by the *phn* operon. APRTase is arbitrarily chosen among the 10 phosphoribosyltransferases of *E. coli* [21]. Any of these 10 enzymes may participate in the process. The pathway is established on the basis of refs. 4, 5, 6, 7, 11 and 13, as well as results of the present work.

The catabolism of phosphonates by the C–P lyase pathway involves a number of enzymatic activities and intermediates the latter of which are ribose derivatives analogous those shown in Figure 1 [7], [11], [12]. *In vitro* analysis has shown that initially a phosphonate moiety displaces adenine of ATP with the formation of a phosphate ester and with inversion of the configuration of the anomeric carbon. The product is 5′-triphospho-α-d-ribosyl 1′-phosphonate and the reaction presumably is catalyzed by PhnI, and, in some way assisted by PhnG, PhnH and PhnK or PhnL. Next the α,β-diphosphoryl bond of 5′-triphospho-α-d-ribosyl 1′-phosphonate is hydrolyzed by PhnM to generate 5′-phospho-α-d-ribosyl 1′-phosphonate [5]. The latter compound is the substrate for C–P lyase encoded by *phnJ*, the product being 5-phospho-α-d-ribosyl 1,2-cyclic phosphate. Polypeptides other than PhnJ, such as PhnG, PhnH, PhnI, PhnK or PhnL may participate in the C–P bond cleavage as well [5], [7]. 5-Phospho-α-d-ribosyl 1,2-cyclic phosphate is then hydrolyzed to form α-d-ribosyl 1,5-*bis*phosphate, a reaction catalyzed by *phnP*-specified phosphoribosyl cyclic phosphodiesterase [7]. The fate of the phosphonate-derived phosphorus (*i.e.* that of the 1-phosphate of α-d-ribosyl 1,5-*bis*phosphate) is presently unknown, but it has been shown that *phnN*-specified ribosyl *bis*phosphate phosphokinase phosphorylates α-d-ribosyl 1,5-*bis*phosphate to 5-phospho-α-d-ribosyl 1-diphosphate (PRPP) [6]. Phosphonate phosphorus would end up as P_i_ following the activities of phosphoribosyltransferases, which catalyze the general reaction PRPP+nitrogenous base→PP_i_+ribonucleoside 5′-monophosphate, and inorganic diphosphatase, which hydrolyzes PP_i_ to P_i_ [6]. Two of the aforementioned intermediates also appear in the growth medium of *phnP* strains as the dephosphorylated derivatives α-d-ribosyl 1′-phosphonate and α-d-ribosyl 1,2-cyclic phosphate (Rib1,2cP). Reactions that are responsible for the formation of P_i_ from organophosphonate via the C–P lyase pathway with AmMePn as an example are given in Figure 1. Finally, a multi-subunit complex consisting of PhnGHIJK has been identified and has been proposed to form the active C–P lyase in *E. coli*. This protein complex may be responsible for the reactions labeled PhnI* and PhnJ* in Figure 1 [4]. The catabolism of AmMePn and other aminoalkylphosphonates has been studied in *E. coli*. The fate of the phosphorus atom is described above. The aminomethyl moiety of AmMePn is found in the growth medium as *N*-methylacetamide. Similarly, the catabolism of *N*-methylaminomethylphosphonate results in the formation of *N,N*-dimethylacetamide [13]. Thus, the compounds at some point during catabolism must be acetylated, presumably by *phnO*-specified aminoalkylphosphonate *N*-acetyltransferase. By analogy, the catabolism of both 1-aminoethylphosphonate and 2AmEtPn would generate *N*-ethylacetamide.

In the present work we analyzed the physiological importance of the *E. coli phnO* gene. We show that the *phnO* gene encodes aminoalkylphosphonate *N*-acetyltransferase, and that this enzyme is essential for the utilization of 1-aminoalkylphosphonates (AmMePn and (*S*)-1-aminoethylphosphonate (S1AmEtPn)) as P_i_ source by the C–P lyase pathway, and that the enzyme is essential for detoxification of the d-alanine analog S1AmEtPn. Additionally, the alleged substrate for C–P lyase, 5′-phospho-α-d-ribosyl 1′-(2-*N*-acetamidoethylphosphonate) (5′PRib1′2NAcAmEtPn), was identified in a supernatant of 2AmEtPn-grown cells of a *phnP* strain.

## Results

### *N*-Acetyltransferase activity of the *phnO* gene product

**Figure 2:**
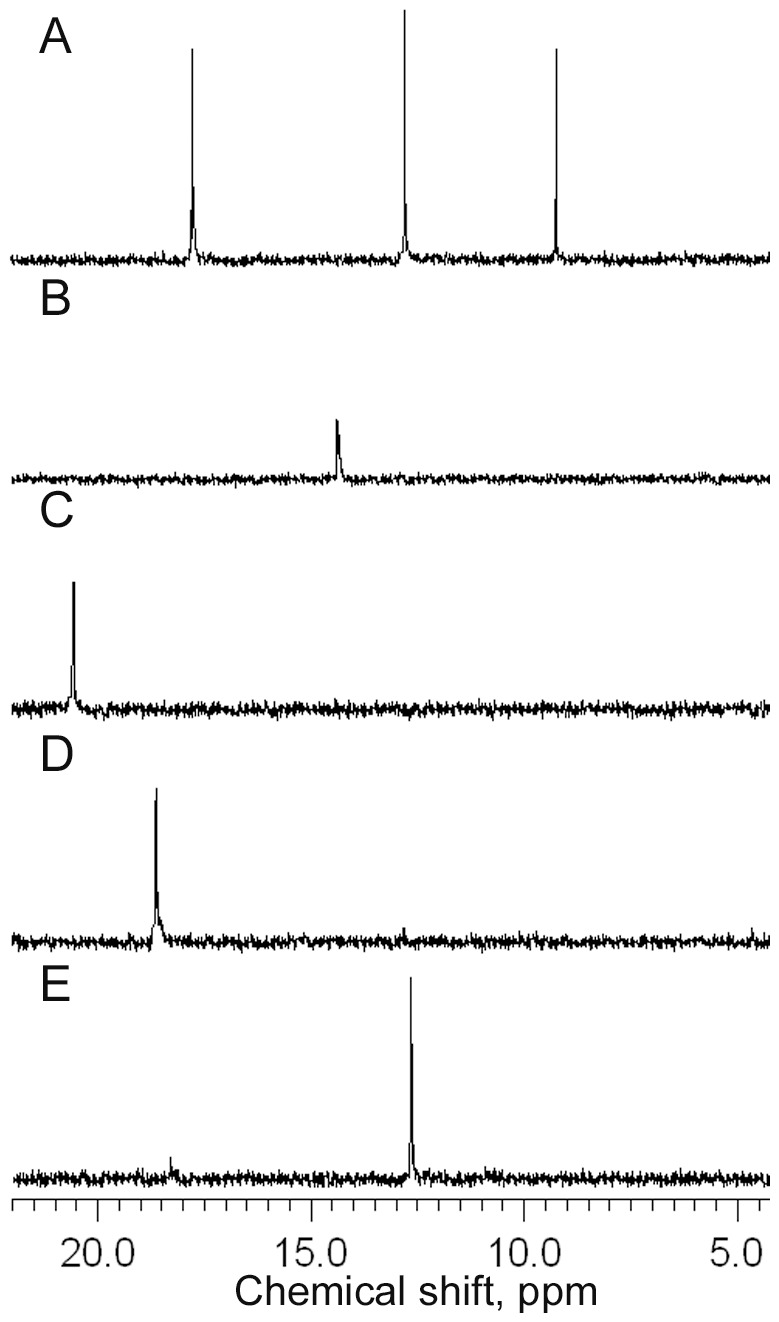
^31^P NMR spectra of reactions with purified aminoalkylphosphonate *N*-acetyltransferase. (A) Reaction mixture without enzyme containing AmMePn (δ 9.2 ppm), 2AmEtPn (δ 17.7 ppm), R1AmEtPn (δ 12.7 ppm) and S1AmEtPn (12.7 ppm). The ^31^P NMR chemical shifts of acetyl coenzyme A (not shown) were δ 1.7 ppm (s), δ −10.7 ppm (d, *J* = 19 Hz) and δ −11.5 ppm (d, *J* = 19 Hz). (B) Reaction product (14.3 ppm) after incubation of enzyme, acetyl coenzyme A and AmMePn, (C) reaction product (δ 20.5 ppm) after incubation of enzyme, acetyl coenzyme A and 2AmEtPn, (D) reaction product (δ 18.3 ppm) after incubation of enzyme, acetyl coenzyme A and S1AmEtPn, (E) reaction product (δ 18.3 ppm) after incubation of enzyme, acetyl coenzyme A and R1AmEtPn.

The amino acid sequences specified by *phnO* of *S. enterica* and by *phnO* of *E. coli* are 77% identical despite their location within different reaction pathways. The two genes, therefore, very likely specify gene products with identical function. To analyze this, histidine-tailed PhnO of *E. coli* was purified by Ni-chelate chromatography and enzymatic activity was determined with AmMePn, 2AmEtPn, (*R*)-1-aminoethylphosphonate (R1AmEtPn) and S1AmEtPn as acetyl receptors and acetyl coenzyme A as acetyl donor. The reactions were followed by ^31^P nuclear magnetic resonance (NMR) spectroscopy (Figure 2A). The enzyme readily converted AmMePn, S1AmEtPn and 2AmEtPn to products with signals at δ 14.3, 18.1 and 20.5 ppm, respectively (Figure 2B–D), whereas R1AmEtPn sluggishly was converted to a product at δ 18.1 ppm (Figure 2E). Approximately 20% of R1AmEtPn was consumed in one hour under the assay conditions used. Prolonged incubation or addition of more enzyme resulted in additional conversion of R1AmEtPn. *N*-(Methylphosphono)glycine (glyphosate), was not a substrate for aminoalkylphosphonate *N*-acetyltransferase (data not shown). The structure of *N*-acetylated 2AmEtPn, 2-*N*-acetamidoethylphosphonate (2NAcAmEtPn) was confirmed by ^1^H, ^13^C, ^1^H/^13^C heteronuclear single quantum coherence (HSQC) and ^1^H/^13^C heteronuclear multiple bond correlation (HMBC) NMR spectroscopy, see below.

### Aminoalkylphosphonates as P_i_ source for *E. coli*

The growth response to various organophosphonates of strains containing the *phnO38* or *ΔphnO789* alleles was compared to that of *phn^+^* and *Δphn* strains (Table 1). A *phn^+^* strain (HO3414) utilized AmMePn, 2AmEtPn and 3AmPrPn as well as MePn, EtPn and PrPn as P_i_ source. The two *phnO* strains utilized 2AmEtPn and 3AmPrPn as well as MePn, EtPn and PrPn as P_i_ source, whereas they were unable to utilize AmMePn. Although the two *phnO* strains qualitatively responded similarly to the various phosphonates, the growth of strain HO3413 (*ΔphnO789*) in general was poorer than that of strain BW17572 (*phnO38*). AmMePn may be regarded as an analog of glycine and could be expected to take the place of glycine in one or more biochemical reactions. However, the addition of glycine to AmMePn-containing medium did not restore growth of the *phnO* strains (not shown). In contrast, the addition of P_i_ to AmMePn-containing medium restored growth. A *Δphn* strain (HO2678) as expected was unable to utilize any of the phosphonates tested. We conclude from these observations that the *phnO*-specified aminoalkylphosphonate *N*-acetyltransferase is obligatory for growth with AmMePn as P_i_ source, but not for growth with the other aminoalkylphosphonates 2AmEtPn and 3AmPrPn, nor for growth with alkylphosphonates as P_i_ source.

**Table 1:** Growth response of *phnO* strains to various aminoalkyl- and alkylphosphonates.

		Growth with phosphate source^a^								
Strain	Lesion	None	AmMePn	AmMePn+P_i_	2AmEtPn	3AmPrPn	MePn	EtPn	PrPn	P_i_
BW17572	*phnO38*	−	−	+++	+++	++	+++	++	+	+++
HO3413	*ΔphnO789*	−	−	+++	++	+	++	++	+	+++
HO3414	*phn^+^*	−	++	+++	+++	++	+++	++	+	+++
HO2578	*Δphn33-30*	−	−	+++	−	−	−	−	−	+++

a Growth was recorded after 48 h of incubation at 37°C: −, no growth; +and ++, intermediate growth; +++, normal (wild-type-like) growth. Phosphorus-containing compounds were added at a concentration of 0.3 mM.

The utilization of the *S* and *R* enantiomers of 1-aminoethylphosphonate was also analyzed. To test if S1AmEtPn was used as a P_i_ source, special conditions were employed. This compound was found to be conditionally bacteriocidal for several of the strains used, as cell lysis was initiated approximately 20 min upon addition of the compound to cell suspensions. S1AmEtPn is an analog of d-alanine, a precursor of bacterial peptidoglycan biosynthesis, and as such is a competitive inhibitor of alanine racemase [14]. To overcome this inhibitory effect, cultures of cells grown with S1AmEtPn were supplemented with d-alanine (or in some cases d,l-alanine), which rescued the cells from lysis. Cell lysis, therefore, was caused by inhibition of cell wall synthesis. A typical growth curve of strain HO2568 (*phn^+^ ΔpstS*) grown with or without d,l-alanine is shown in Figure S1. The addition of S1AmEtPn caused rapid lysis of the culture without d,l-alanine, whereas the addition of S1AmEtPn appeared to induce only a temporary arrest in growth of the culture with d,l-alanine present. Interestingly, strain HO2680 (*Δphn ΔpstS*) also lysed in the presence of S1AmEtPn (not shown). HO2680 is unable to transport phosphonate by the cognate phosphonate transport system, suggesting that at least under these conditions the compound was taken up by a different transport system, which was not the high affinity P_i_ transport system specified by *pstSCAB*, as this was also deleted in the strain. The growth response of the various strains to 1-aminoethylphosphonates in solid medium is shown in Table 2. None of the strains grew with S1AmEtPn as sole supplement. If P_i_ was added in addition to S1AmEtPn the *phn^+^* strain grew, whereas the *phnO* strains (and also the *Δphn* strain) formed very small colonies, which appeared heterogeneous in colony size and morphology, thus, demonstrating the toxic effect of S1AmEtPn described above. When P_i_ was supplied none of the strains needed to utilize S1AmEtPn. However, the wild-type strain was able to acetylate S1AmEtPn, and, thus, presumably detoxify the compound, whereas the *phnO* strains were unable to acetylate and detoxify the compound, and, consequently, no or poor growth occurred. When d-alanine was added in addition to S1AmEtPn the *phn^+^* strain grew, whereas the *phnO* strains did not. Here d-alanine competed with its analog S1AmEtPn and inhibition was overcome. In contrast, the *phnO* strains were unable to grow even though d-alanine presumably also overcame the inhibition by S1AmEtPn in these strains. Therefore, the reason for lack of growth of the *phnO* strains was a lack of acetylation, and, thus, acetylation is a requisite also for catabolism of S1AmEtPn. With addition of both d-alanine and P_i_, in addition to S1AmEtPn, the *phnO* strains resumed growth. Again d-alanine overcame the inhibition by S1AmEtPn, and the presence of P_i_ made the presence of S1AmEtPn redundant as P_i_ source. The reason for lack of growth of strain HO3414 (*phn^+^*) with S1AmEtPn as sole supplement is presently not clear. Constitutive expression of the *phn* operon, and, thus, of the *phnO* cistron was not sufficient to obtain growth with S1AmEtPn as sole P_i_-source, as strain HO3417 (*phn^+^ ΔpstS*) responded similarly to strain HO3414 (*phn^+^ pstS^+^*).

**Table 2:** Growth response of *phnO* strains to 0.3 mM 1-aminoethylphosphonate.^a^

		Growth with					
		S1AmEtPn and additive(s)				R1AmEtPn ± P_i_	
Strain	Lesion	None	P_i_	d-Alanine	P_i_+d-alanine	−P_i_	+P_i_
BW17572	*phnO38*	−	−b	−	+++	−	+++
HO3413	*ΔphnO738*	−	−b	−	+++	−	+++
HO3414	*phn^+^*	−	+++	++	+++	−	+++
HO2678	*Δphn*	−	−b	−	+++	−	+++

a Growth conditions and recording were those described in Table 1. The concentration of d-alanine was 100 mg L^−1^.

b Few very small colonies, heterogeneous in size and morphology.

The effect on cell growth of R1AmEtPn was much less dramatic. Although R1AmEtPn inhibited the growth of *phn^+^* strains, such as HO2568 and HO3414, no lysis was observed. This inhibition could not be alleviated neither by the addition of l-alanine, of which R1AmEtPn may be an analog, nor by the addition of d-alanine or both. Neither the *phn^+^* nor the *phnO* strains utilized R1AmEtPn as P_i_ source. Furthermore, all of the *phn^+^* and *phnO* strains grew when P_i_ was added together with R1AmEtPn, showing that R1AmEtPn probably did not exert any severe toxic effect. We conclude from these observations that R1AmEtPn is not a P_i_-source for *E. coli*. This observation may be consistent with R1AmEtPn being a poor substrate for *phnO*-specified aminoalkylphosphonate *N*-acetyltransferase.

### Accumulation of 2AmEtPn catabolic-intermediates in cultures of *E. coli phn* mutant strains

**Figure 3:**
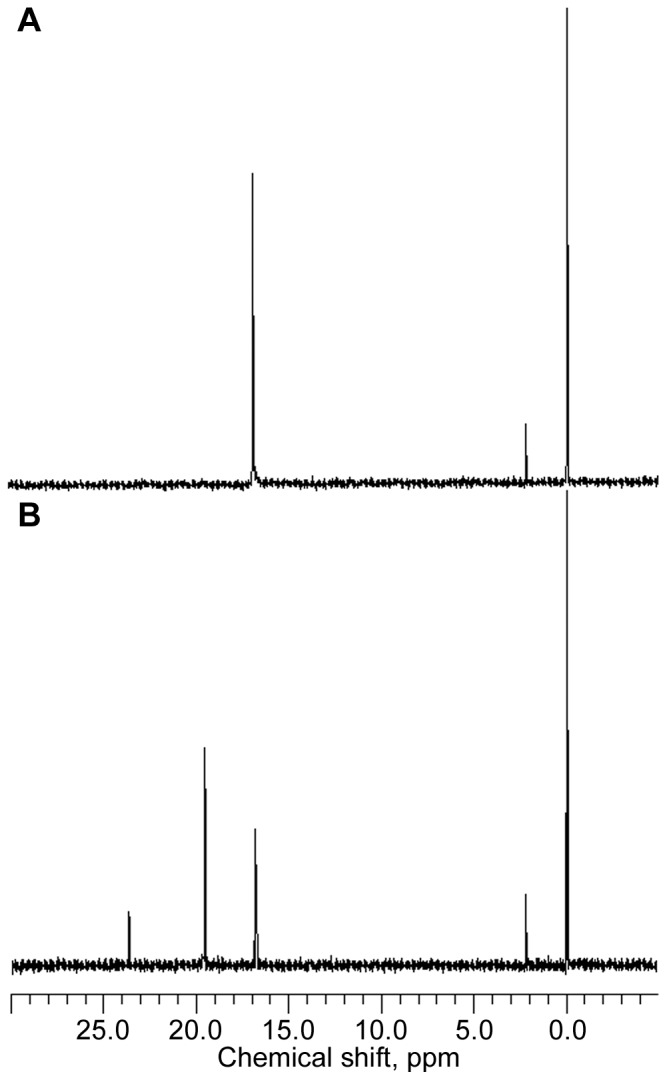
Conversion of 2AmEtPn by a *phn^+^* strain analyzed by ^31^P NMR. Cells of strain HO2568 (*phn^+^ ΔpstS*) were grown in 03P medium in the presence of 2AmEtPn and supernatant fluids were analyzed as described in Materials and Methods. (A) ^31^P NMR spectrum of culture supernatant immediately after addition of 2AmEtPn. Chemical shifts: 16.8 ppm, 2AmEtPn; 2.3 ppm P_i_. (B) ^31^P NMR spectrum of culture supernatant after 20 h of incubation with 2AmEtPn. Chemical shifts: 23.6 ppm, Rib1′N2AcAmEtPn; 19.4 ppm, 2NAcAmEtPn; 2.3 ppm P_i_. The peak at 0.0 ppm represents the external standard.

**Figure 4:**
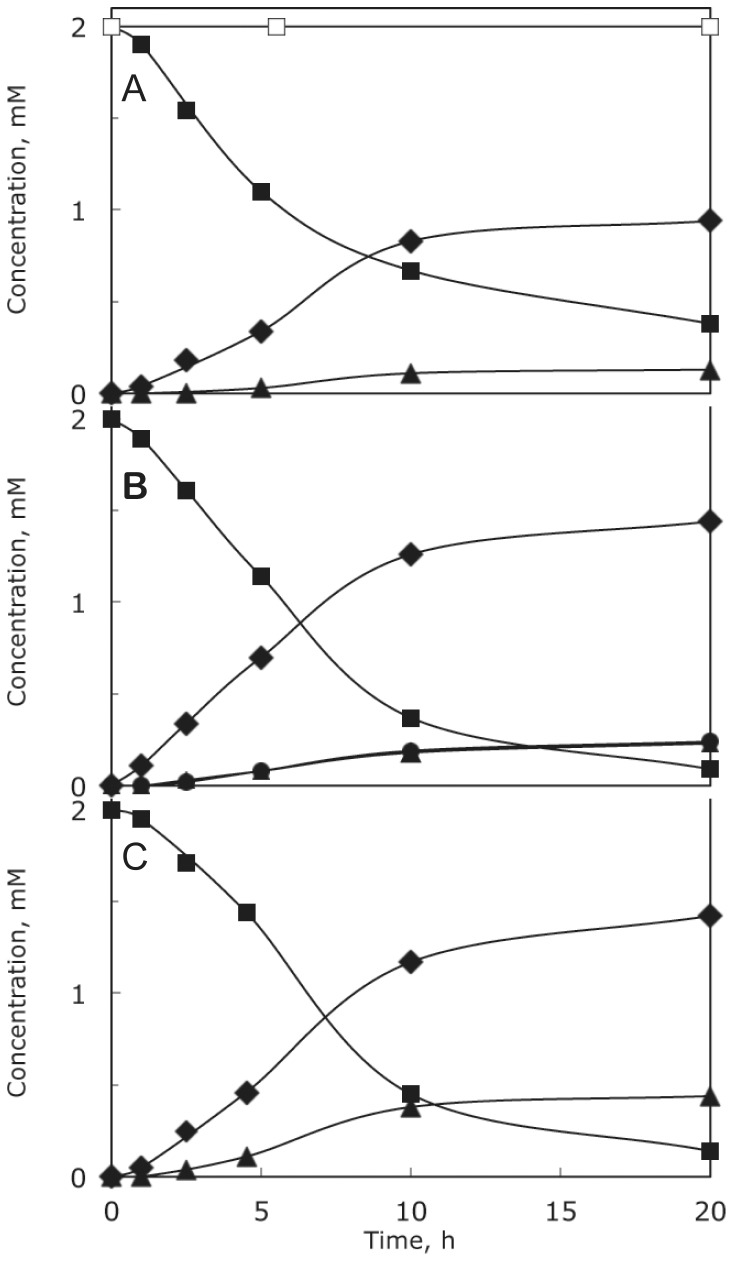
Time course of conversion of 2AmEtPn by *phn^+^*, *phnO*, *phnP* and *phnJ* strains. Symbols: squares, 2AmEtPn (δ 16.8 ppm); diamonds, 2NAcAmEtPn (δ 19.4 ppm); triangles, Rib1′N2AcAmEtPn (δ 23.6 ppm); circles, Rib1,2cP (δ 18.6 ppm). (A) Strain HO2568 (*phn^+^ ΔpstS*) (closed symbols) and strain HO2541 (*phnO38 ΔpstS*) (open symbol); (B) strain HO2542 (*phnP ΔpstS*); (C) strain HO2536 (*phnJ ΔpstS*).

The *phn* mutant strains employed in this analysis contained the *ΔpstS605* allele, which served to render *phn* operon expression constitutive and, thus, independent of the phosphate supply. The conversion of aminoalkylphosphonate and the accumulation of intermediates of aminoalkylphosphonate catabolism were analyzed by ^31^P NMR spectroscopy of the growth medium, which also contained P_i_, as several of the *phn* mutants used were unable to use phosphonate as phosphate source. A *phn^+^ ΔpstS* strain (HO2568) was grown in the presence of 2AmEtPn. Figure 3A shows a ^31^P NMR spectrum of the culture medium immediately after the addition of 2AmEtPn (δ 16.8 ppm), whereas Figure 3B shows a ^31^P NMR spectrum of the culture medium after 20 h of incubation at 37°C. Here the δ 16.8 ppm-peak is greatly diminished, and, in addition, two additional peaks appear (δ 19.5 and 23.6 ppm). The two compounds were 2NAcAmEtPn (19.5 ppm) and α-d-ribosyl 1′-(*N*-2-acetamidoethylphosphonate) (Rib1′N2AcEtPn) (23.6 ppm). The time course is shown in Figure 4A (closed symbols). 2AmEtPn (δ 16.8 ppm) disappeared quite rapidly, and the δ 19.5 ppm-compound similarly was formed quite rapidly, whereas the 23.6 ppm-compound appeared only sluggishly. The loss of phosphonate-phosphorus to the cells, which were removed before NMR analysis, was approximately 20% over the 20 h-incubation. This loss of phosphonate-phosphorus originates from conversion to P_i_ followed by incorporation into nucleic acids. Results of a similar experiment performed with a *phnO ΔpstS* strain (HO2541) were remarkably different (Figure 4A, open squares). There was no acetylation at all of 2AmEtPn, which demonstrates that the formation of the compounds of δ 19.5 and 23.6 ppm was dependent on the *phnO* gene product. A summary of the various ^31^P NMR chemical shifts obtained and their assignments are given in Table 3.

**Table 3:** Summary of ^31^P NMR chemical shifts observed in culture supernatants of various *phn* strains grown with various organophosphonates.^a^

Strain	Lesion	Addition	Chemical shift (ppm)	Assignment
HO2568	*phn^+^*	AmMePn	9.2	AmMePn
			13.9^b^	NAcAmMePn
			17.4	Rib1′NAcAmMePn
		2AmEtPn	16.8	2AmEtPn
			19.4^c^	2NAcAmEtPn
			23.6	Rib1′N2AcAmEtPn
		R1AmEtPn	12.6	R1AmEtPn
		S1AmEtPn	12.6	S1AmEtPn
			18.1^d^	S1NAcAmEtPn^e^
			20.6	Rib1′S1NAcAmEtPn^f^
		EtPn	27.5	EtPn
		MePn	23.9	MePn
		PrPn	25.8	PrPn
HO2541	*phnO38*	AmMePn	9.2	AmMePn
		2AmEtPn	16.8–17.0	2AmEtPn
		S1AmEtPn	12.6	S1AmEtPn
		EtPn	27.5	EtPn
HO3412	*ΔphnO789*	AmMePn	9.2	AmMePn
		2AmEtPn	17.0	2AmEtPn
			18.6	Rib1,2cP
		S1AmEtPn	12.6	S1AmEtPn
HO2542	*phnP*	AmMePn	9.2	AmMePn
			13.9–14.0	NAcAmMePn
			17.4	Rib1′NAcAmMePn
			18.6	Rib1,2cP
		2AmEtPn	16.8–17.1	2AmEtPn
			18.6	Rib1,2cP
			19.6–20.2	2NAcAmEtPn
			23.6	Rib1′N2AcAmEtPn
		R1AmEtPn	12.6	R1AmEtPn
		S1AmEtPn	12.6	S1AmEtPn
			18.0	S1NAcAmEtPn^e^
			18.6	Rib1,2cP
			20.7	Rib1′S1NAcAmEtPn^f^
		EtPn	18.6	Rib1,2cP
			27.6	EtPn
			30.3	Rib1′EtPn^g^
HO2536	*phnJ*	AmMePn	9.2	AmMePn
			13.9	NAcAmMePn
			17.4	Rib1′NAcAmMePn
		2AmEtPn	16.9–17.0	2AmEtPn
			19.7–20.0	2NAcAmEtPn
			23.6	Rib1′N2AcAmEtPn
		EtPn	27.6	EtPn
			30.3	Rib1′EtPn

a Data are from samples taken after 20 h of incubation in the presence of phosphonate. Due to uneven acidification of the growth media chemical shifts occasionally showed small differences among otherwise similar cultures. Particularly 2NAcAmEtPn was prone to pH dependent chemical shifts (up to 0.6 ppm).

b This signal was assigned as NAcAmMePn by spiking the NMR sample with the reaction product of aminoalkylphosphonate *N*-acetyltransferase with AmMePn as the acetyl acceptor.

c This signal was assigned as 2NAcAmEtPn by spiking the NMR sample with the reaction product of aminoalkylphosphonate *N*-acetyltransferase with 2AmEtPn as the acetyl acceptor.

d This signal was assigned as S1NAcAmEtPn by spiking the NMR sample with the reaction product of aminoalkylphosphonate *N*-acetyltransferase with S1AmEtPn as the acetyl acceptor.

e S1NAcAmEtPn, *N*-acetyl-(*S*)-1-aminoethylphosphonate.

f Rib1′S1NAcAmEtPn^f^, α-d-ribosyl 1′-(*N*-acetyl-(*S*)-1-aminoethylphosphonate).

We furthermore analyzed the fate of 2AmEtPn in the culture medium of a *phnP ΔpstS* strain (HO2542), Figure 4B. Here 2AmEtPn was converted to three compounds, two of which were the same as those formed by the *phn^+^* strain, δ 19.5 and 23.6 ppm, and a third compound with δ 18.6 ppm. The latter peak was caused by Rib1,2cP, which is a prominent phosphonate-catabolic intermediate of *phnP* strains [7]. Analysis of a *phnJ ΔpstS* strain (HO2536) revealed a pattern similar to that of the *phnP* strain, except that Rib1,2cP was not formed as evidenced by a lack of the δ 18.6 ppm-peak, which was expected as the *phnJ* gene product exerts its function (C–P bond cleavage) before that of the *phnP* gene product (cyclic phosphodiester hydrolysis) [7], Figure 4C. With strains HO2542 and HO2536 there was no loss of phosphonate phosphorus to the cells, which was also expected, as these strains are unable to convert phosphonate to a usable phosphorus-containing compound.

### Chemical structure of the 19.5 ppm-compound, 2NAcAmEtPn

**Figure 5:**
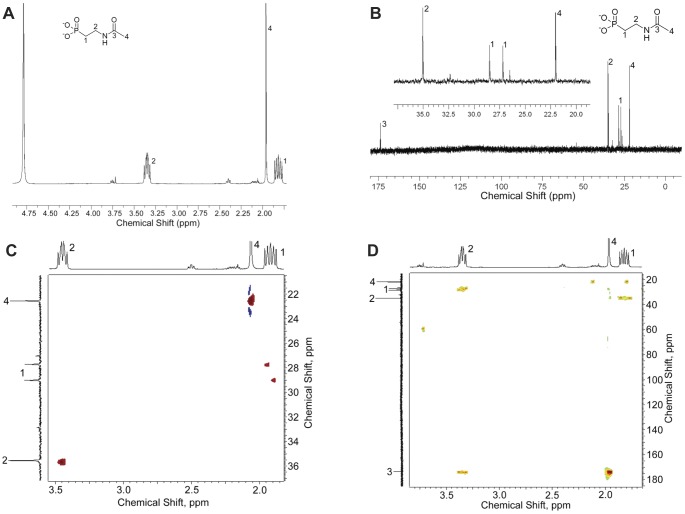
Characterization by NMR of 2NAcAmEtPn. Spectra: A, ^1^H NMR; B, ^31^C NMR; C, ^1^H/^13^C HSQC NMR; D. ^1^H/^13^C HMBC NMR.

**Figure 6:**
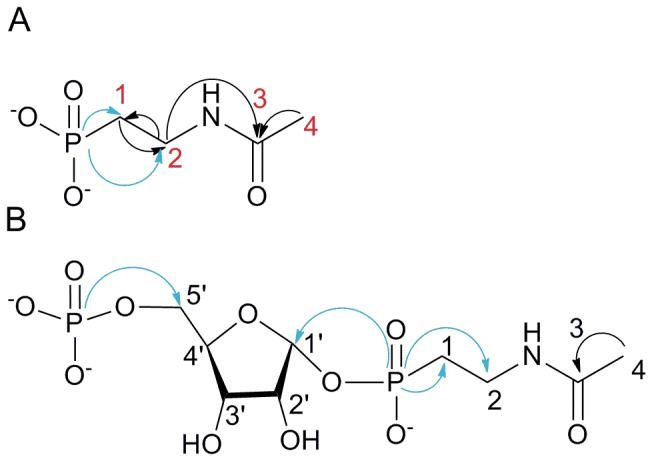
Observed ^1^H/^13^C HSQC, ^1^H/^13^C HMBC and ^1^H/^31^P HMBC correlations of 2NAcAmEtPn and 5′PRib1′2NAcAmEtPn. Black arrows: correlations observed by ^1^H/^13^C HSQC and ^1^H/^13^C HMBC spectroscopy; blue arrows: correlations observed by ^1^H/^31^P HMBC spectroscopy. A, 2NAcAmEtPn; B, 5′PRib1′2NAcAmEtPn.

The supernatant of a 2AmEtPn-grown culture of strain HO2542 (*phnP*) was applied to an ion-exchange column, and the various phosphorus-containing compounds were separated (Materials and Methods). Elution was followed by ^31^P NMR spectroscopy, Figure S2A. The structure of the compound responsible for the δ 19.5 ppm-peak was shown by ^1^H, ^13^C and ^31^P NMR spectroscopy to be 2NAcAmEtPn. The following signals were obtained: ^1^H NMR (400 MHz, D_2_O) δ ppm 3.35 (doublet (d) of triplets (t), coupling constant (*J*) = 7.6, 7.6, 10.2 Hz, 2H), 1.96 (singlet (s), 3H), 1.82 (multiplet (m), *J* = 7.6, 7.6, 16.1 Hz, 2H) (Figure 5A); ^13^C NMR (101 MHz, D_2_O) δ ppm 173.89 (s, 1CO), 35.04 (s, 1CH_2_), 27.85 (d, *J_CP_* = 131 Hz, 1CH_2_), 22.03 (s, 1CH_3_) (Figure 5B). Protons were assigned to carbons by ^1^H/^13^C HSQC NMR (Figure 5C), and by ^1^H/^13^C HMBC NMR (Figure 5D). ^1^H/^13^C correlations from HSQC and HMBC NMR spectra are shown in Figure 6A. Furthermore, 2NAcAmEtPn was also the product of aminoalkylphosphonate *N*-acetyltransferase activity with 2AmEtPn as substrate. This was demonstrated by mixing the two compounds (*i.e.* reaction product and supernatant fluid), which resulted in enlargement of a single peak (^31^P NMR, δ 19.5 ppm) rather than formation of two individual peaks (data not shown).

### Accumulation of AmMePn catabolic-intermediates in cultures of *E. coli phn* mutant strains

**Figure 7:**
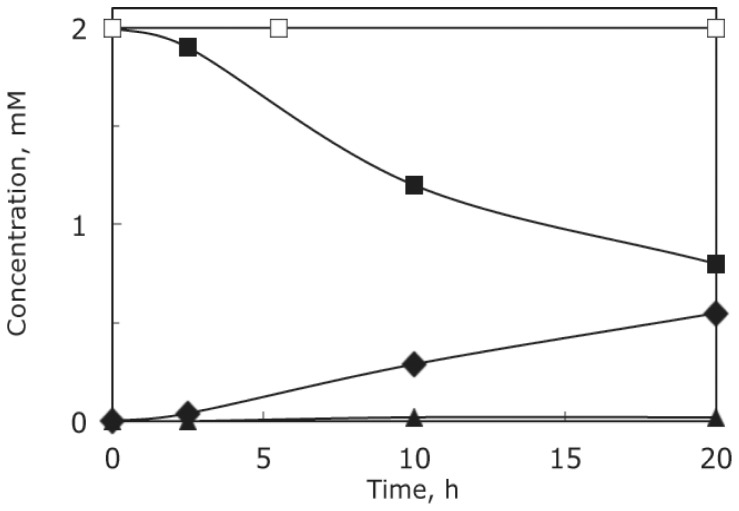
Time course of conversion of AmMePn by strain HO2568 (*phn^+^*) (closed symbols) and strain HO2541 (*phnO38*) (open symbol). Symbols: squares, AmMePn (δ 9.2 ppm); diamonds, NAcAmMePn (δ 13.9 ppm); triangles, Rib1′NAcAmMePn (δ 17.4 ppm).

The conversion of AmMePn was also analyzed. In general, the same pattern emerged. AmMePn was acetylated and further catabolized by the *phn^+^*, the *phnJ* and the *phnP* strain, and compounds with chemical shifts δ 13.9 and 17.4 ppm appeared in cultures of all three strains (in addition to residual AmMePn of δ 9.2 ppm). The two new compounds were *N*-acetamidomethylphosphonate (NAcAmMePn) (13.9 ppm) and α-d-ribosyl 1′-(*N*-(acetamidomethylphosphonate) (Rib1′NAcAmMePn) (17.4 ppm). As expected the compound of δ 18.6 ppm (Rib1,2cP) also appeared in the AmMePn-fed cells of the *phnP* strain. The response, however, was less dramatic than that with 2AmEtPn. As with 2AmEtPn, there was no acetylation at all of AmMePn by the *phnO* strains, Figure 7, Table 3. The response of strains HO2542 (*phnP*) and HO2536 (*phnJ*) to AmMePn is shown in Figure S3 and S4, respectively. The more sluggish disappearance of AmMePn, as compared to that of 2AmEtPn, is consistent with the poorer growth of strain HO2568 (*phn^+^*) with AmMePn as P_i_ source compared to that with 2AmEtPn (Table 1).

The accumulation of compounds in the used medium after growth of the four strains HO2568 (*phn^+^ ΔpstS*), HO2541 (*phnO ΔpstS*), HO2536 (*phnJ ΔpstS*), and HO2542 (*phnP ΔpstS*) in the presence of EtPn for 20 h was also analyzed. Strains HO2536 and HO2542 contained a compound with a chemical shift δ 30.3 ppm in addition to remaining EtPn (δ 27.6 ppm), whereas there were no phosphorus-containing compounds other than EtPn in the supernatant fluids of strains HO2568 and HO2541. The δ 30.3 ppm-compound has been previously detected and determined as α-d-ribosyl 1′-ethylphosphonate (Rib1′EtPn) [7]. As expected, the *phnP* strain also accumulated Rib1,2cP, a substrate of *phnP-*specified phosphoribosyl cyclic phosphodiesterase (Table 3) [7].

Although *phnO^+^* strains readily acetylated 2AmEtPn to form 2NAcAmEtPn the process apparently was not necessary for catabolism of 2AmEtPn as demonstrated by the ability of *phnO* mutant strains to utilize 2AmEtPn as a P_i_ source (Table 1). A parallel process by which 2-aminoalkylphosphonate is catabolized in the absence of acetylation was also demonstrated, as the *ΔphnO789*-harboring strain (HO3412) accumulated Rib1,2cP when fed 2AmEtPn (Table 3). In contrast, when strain HO3412 was fed AmMePn no Rib1,2cP could be detected in the culture supernatant, providing support for the suggestion that the catabolism of 1-aminoalkylphosphonates requires acetylation.

### Accumulation of 5′PRib1′2NAcAmEtPn in the culture of a 2AmEtPn-grown *phnP* strain

**Figure 8:**
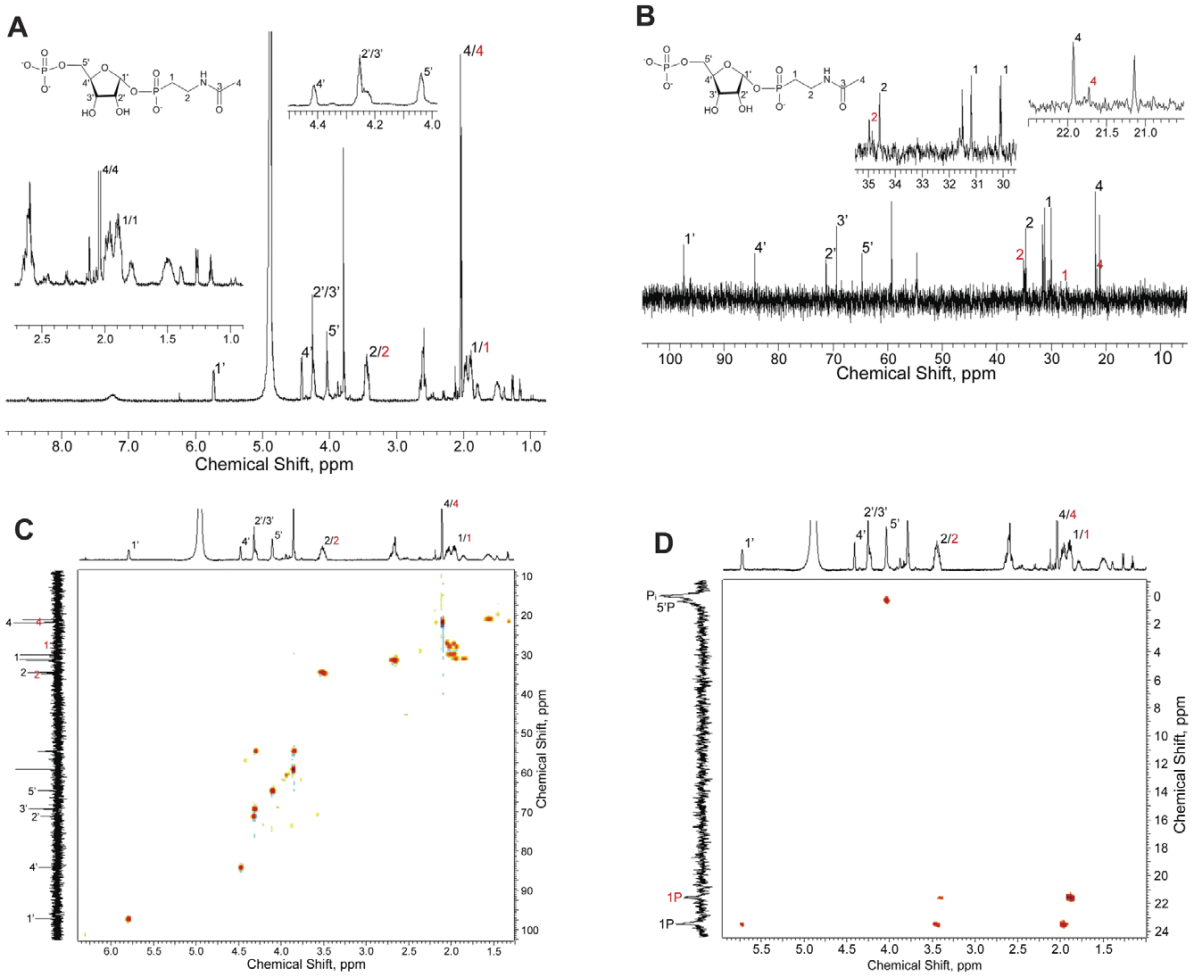
Characterization by NMR of 5′PRib1′2NAcAmEtPn. Protons, carbons and phosphorus of 2NAcAmEtPn are labeled in red. Spectra: A, ^1^H NMR; B, ^13^C NMR; C, ^1^H/^13^C HSQC NMR; D, ^1^H/^31^P HMBC NMR.

During the purification of 2NAcAmEtPn by ion-exchange chromatography we noticed the elution of a compound with chemical shifts δ 24 and 3.6 ppm by ^31^P NMR spectroscopy. The chemical shift values suggested a structure containing two phosphorus atoms, one of which was bound to carbon (δ 24 ppm) the other being a phosphate ester (δ 3.6 ppm) (Figure S2B). Selected fractions were concentrated and characterized by NMR spectroscopy. Although the preparation also contained 2NAcAmEtPn, we unequivocally identified the presence of 5′PRib1′2NAcAmEtPn. Protons were assigned to carbons on the basis of ^1^H/^1^H correlation spectroscopy (COSY), ^13^C, ^13^C-distortionless enhancement by polarization transfer (DEPT) NMR, ^1^H/^13^C HSQC and ^1^H/^13^C HMBC NMR spectra as well as ^1^H/^31^P HMBC NMR spectra. The following signals were obtained: ^1^H NMR (600 MHz, D_2_O): δ 5.73 (H1′, m, 1H), 4.41 (H4′, m, 1H), 4.24 (H2′/H3′, m, 3H), 4.04 (H5′, m, 2H), 3.45 (H2 of 5′PRib1′2NAcAmEtPn and 2NAcAmEtPn, m, 4H), 2.04 (H4 of 5′PRib1′2NAcAmEtPn and 2NAcAmEtPn, m, 5H), 1.89 (H1 of 5′PRib1′2NAcAmEtPn and 2NAcAmEtPn as well as a contaminating compound, m, 7H) (Figure 8A); ^13^C NMR (125 MHz, D_2_O) δ 173.9 (s, CO, C3), 97.4 (d, *J*_CP_ = 5.8 Hz, CH, C1′), 84.3 (d, *J*_CP_ = 8.5 Hz, CH, C4′), 71.3 (s, CH, C2′), 69.4 (s, CH, C3′), 64.7 (d, *J*_CP_ = 4.6 Hz, CH_2_, C5′), 34.6 (s, CH_2_, C2), 30.6 (d, *J*_CP_ = 137 Hz, CH_2_, C1), 22.0 (s, CH_3_, C4) (Figure 8B); ^31^P NMR (400 MHz, D_2_O) δ 23.7 (P1) and 0.55 (P5), in addition to 21.7 ppm (P of 2NAcAmEtPn) and 0.17 ppm P_i_. The two-dimensional NMR spectra of 5′PRib1′2NAcAmEtPn are shown in Figure 8C (^1^H/^13^C HSQC spectrum) and Figure 8D (^1^H/^31^P HMBC spectrum) as well as Figure S5 (^1^H/^1^H COSY spectrum) and Figure S6 (^1^H/^13^C HMBC spectrum). A list of the chemical shifts of individual protons and their correlation with carbon and phosphorus atoms is given in Table 4. Observed ^1^H/^13^C correlations from HSQC and HMBC NMR spectra as well as ^1^H/^31^P correlations from HMBC NMR spectra are shown in Figure 6B. Finally, analysis by electrospray ionization mass spectrometry (ESI-MS) (negative ion mode) revealed a molecular ion peak at *m*/*z* = 378.0351 corresponding to the expected value for the molecular ion of 5′PRib1′2NAcAmEtPn (C_9_H_18_NO_11_P_2_) (378.0355). Another compound (δ 24 ppm) was also observed eluting 50 to 97 mL. This compound was not characterized further but is believed to be Rib1′N2AcEtPn, *i.e.* a 5′-dephosphorylated derivative of 5′PRib1′2NAcAmEtPn [7].

**Table 4:** Assignment of the NMR spectroscopic signals observed for 5′PRib1′2NAcAmEtPn.^a^

C	^13^C NMR (ppm)	^1^H NMR (ppm, multiplicity, integration)	^1^H/^13^C HMBC (ppm)	^1^H/^31^P HMBC (ppm)
1	30.6	1.89, m, 7^b^		23.5, 21.5
2	34.6	3.45, m, 4		23.5, 21.6
3	173.9			
4	22.0	2.04, s, 5	172.1, 173.9	
1′	97.4	5.73, m, 1		23.5
2′	71.3	4.24, m, 3^c^		
3′	69.4	4.24, m, 3^c^		
4′	84.3	4.41, m, 1		
5′	64.7	4.04, m, 2		0.34
*1* d	*27.7*	*1.89, m, 7* b		*23.5, 21.6*
*2* d	*34.9*	*3.45, m, 4*		*23.5, 21.6*
*3* d	*172*			
*4* d	*21.7*	*2.04, s, 5*	*172.1, 173.9*	

a The preparation also contained 2NAcAmEtPn and small amounts of one or more contaminants. ^13^C and ^1^H NMR spectra were assigned to correlations observed in the COSY, HSQC and HMBC NMR spectra. ^1^H/^13^C and ^1^H/^31^P HMBC NMR correlations are shown.

b 2H of 5′PRib1′2NAcAmEtPn and 2NAcAmEtPn, each, as well as 3H from contamination.

c H2′ and H3′ as well as H from contamination.

d Data for 2NAcAmEtPn.

### Polarity of the *ΔphnO789* allele on *phnP* gene expression

During genetic manipulations we noted that the utilization of alkylphosphonates or aminoalkylphosphonates other than AmMePn as P_i_ source was considerably retarded in strains harboring the *ΔphnO789::kan* allele compared to strains harboring the *phnO38::*Tn*phoA-*9′ or wild-type *phn^+^* alleles. The three strains were isogenic as they differed only with respect to the *phnO* alleles. Nevertheless, strain HO3413 (*ΔphnO789*) consistently formed smaller colonies on solid medium than did strains HO3414 (*phn^+^*) or HO3418 (*phnO38*). This behavior is also evident from the data of Table 1. Compare for example the growth response of strain BW17572 (*phnO38*) with that of strain HO3413 (*ΔphnO789*) on 2AmEtPn as P_i_ source. Additionally, the doubling times in medium containing MePn, which is catabolized without the participation of the *phnO* gene product, as P_i_ source were 180, 160 and 300 min for HO3414 (*phn^+^*), HO3418 (*phnO38*) and HO3413 (*ΔphnO789*), respectively. Insight to the basis of these differences was gained, when a 2AmEtPn-fed culture of strain HO3412 (*ΔphnO789 ΔpstS*) was observed to accumulate Rib1,2cP (^31^P NMR, δ 18.6 ppm, Table 3) to an amount of approximately 15% of the remaining 2AmEtPn. In contrast, a 2AmEtPn-fed culture of strain HO2541 (*phnO38 ΔpstS*) did not accumulate Rib1,2cP. Rib1,2cP is a substrate of *phnP*-specified phosphoribosyl cyclic phosphodiesterase [7]. Thus, the phosphonate-bradytrophic (*i.e.* slow-growing) phenotype of *ΔphnO789::kan*-harboring strains is ascribed to insufficient activity of *phnP*-specified phosphoribosyl cyclic phosphodiesterase due to polarity of the *ΔphnO789* allele on the expression of the downstream *phnP* cistron.

## Discussion

Acetylation of aminoalkylphosphonates is an efficient process in *E. coli*. A culture of a *phn^+^* strain, such as HO2568, which expressed the *phn* operon constitutively, removed essentially all of the added 2AmEtPn (2 mM) in approximately 24 h. Thus, acetylation of 2AmEtPn by far exceeds the phosphate-need, which is less than 0.4 mM. The efficiency of the process may be related to the detoxification-effect. Acetylation of S1AmEtPn, *i.e.* detoxification, must be efficient to prevent cell-lysis. It is possible, that phosphonate is taken up at a rate higher than that of the catabolism in order to keep the catabolic machinery saturated. With S1AmEtPn, non-acetylated compound would be detrimental to the cell and to prevent this, S1AmEtPn is efficiently acetylated to SNAc1AmEtPn and some of this compound is catabolized, whereas the surplus is released to the growth medium. This overwhelming quantitative acetylation-process, furthermore, suggests that acetylation precedes C–P bond cleavage. The fact that this, indeed, is the case was confirmed by the isolation of a substrate for C–P lyase in cells grown with 2AmEtPn, as this substrate was acetylated (se further below). Acetylation of aminoalkylphosphonates was catalyzed by *phnO*-specified aminoalkylphosphonate *N*-acetyltransferase. Thus, the *phnO*-deficient strains HO2541 and HO3412 were unable to acetylate 2AmEtPn. Despite of this lack of acetylation, 2AmEtPn was an excellent phosphate source for the *phnO*-deficient strains. Furthermore, AmMePn and S1AmEtPn were readily acetylated *in vitro*, and served as P_i_ source for *phnO^+^* strains *in vivo*, but not for *phnO* strains. In fact, the most prominent physiological difference between a *phn^+^* and the *phnO* strains was the inability of the *phnO* strains to remove aminoalkylphosphonate from the growth medium, and their lack of growth on 1-aminoalkylphosphonates (*i.e.* AmMePn or S1AmEtPn) as P_i_ source.

It is likely that one or more enzymatic steps of the C–P lyase pathway are hampered by the presence of a free amino group close to the phosphorus atom of the phosphonate group. One possibility for the catabolic stability of AmMePn is the effect of the amino group on the stability of the carbon-centered radical upon C–P bond cleavage. At physiological pH the amino group of AmMePn can be expected to be protonated and have a formal positive charge. It is estimated that the electron withdrawing effect of an ammonium group destabilizes an attached carbon centered radical by 4–5 kcal mol^−1^ [15], [16]. This has been used to explain the regioselectivity of radical formation at carbon atoms remote from the α-amino group of amino acids or the ε-amino group of lysine [17]. Conversely, *N*-acetylation would neutralize this charge, as well as assist radical formation by delocalization of the single electron into the acetyl group. The inductive effect of the ammonium group may also explain why 2AmEtPn can undergo C–P bond cleavage without acetylation by aminoalkylphosphonate *N*-acetyltransferase, since this effect will be weaker when the electron-withdrawing group is placed further away from the site of radical formation.

Acetylation serves an additional role in detoxification. S1AmEtPn, an analog of d-alanine, causes inhibition of alanine racemase and inhibition of peptidoglycan biosynthesis resulting in cell lysis. S1AmEtPn can be supplied to *E. coli* as the suicide compound alaphosphin (l-alanyl-l-1-aminoethylphosphonate). After uptake of alaphosphin by a dipeptide transport system followed by hydrolysis by a didpeptidase S1AmEtPn is formed and exerts its bacteriocidal effect [14]. Our results confirmed the toxic effect of S1AmEtPn on cell wall biosynthesis after addition of the free compound. In a *phn^+^* strain the bacteriocidal effect of S1AmEtPn could be overcome by the addition of P_i_ or d-alanine or both. However, *phnO* strains were much more susceptible to inhibition than *phn^+^* strains. Thus, even with P_i_ or d-alanine present, S1AmEtPn was toxic for the *phnO* strains. Specifically, the fact that the *phn^+^* strain grew in the presence of S1AmEtPn and P_i_, whereas the *phnO* strains did not, demonstrates that the acetylation mediated by aminoalkylphosphonate *N*-acetyltransferase is a requisite for detoxification of S1AmEtPn. Under these conditions S1AmEtPn need not function as P_i_ source. Additionally, the fact that the *phn^+^* strain grew in the presence of S1AmEtPn and d-alanine, whereas the *phnO* strains did not, demonstrates that the acetylation is a requisite for utilization of S1AmEtPn as P_i_ source. Although C–P lyase is widely spread among bacterial species, many *phn* operons lack a *phnO* cistron. An example of these microorganisms is *Pseudomonas stutzeri*. This organism contains two C–P lyase specifying operons (*htx* and *phn*) none of which contain a *phnO* homolog [18]. Whereas 2AmEtPn is an excellent source of P_i_, the growth response of *P. stutzeri* to AmMePn or S1AmEtPn has not been reported, but if the C–P lyase pathway(s) of this organism share properties with that of *E. coli*, we predict that *P. stutzeri* is unable to utilize neither AmMePn nor S1AmEtPn. Alternatively, a *phnO* homolog may be located outside the *htx* and *phn* operons or some unspecific acetyltransferase may participate in detoxification and catabolism of AmMePn and S1AmEtPn in *P. stutzeri*. Furthermore, in C–P lyase-less *S. enterica* aminoalkylphosphonate *N*-acetyltransferase may serve as a detoxifying enzyme as previously suggested [9].

Although our data demonstrates a physiological role of aminoalkylphosphonate *N*-acetyltransferase, acetylation of AmMePn and S1AmEtPn, it remains to be established if AmMePn and S1AmEtPn are naturally produced compounds. Additionally, it is possible that other 1-aminoalkylphosphonates are also substrates for aminoalkylphosphonate *N*-acetyltransferase and that some of these compounds are naturally produced. Indeed, AmMePn is a prominent intermediate in the catabolism of the man-made herbicide glyphosate in soil [19].

By ^32^P-labeling of catabolic intermediates we previously showed that *phnP* strains of *E. coli* accumulate at least two radiolabeled compounds when fed alkylphosphonate or aminoalkylphosphonate. Both compounds contained a radiolabeled phosphate ester and we previously showed that one of these compounds is 5-phospho-α-d-ribosyl 1,2-cyclic phosphate [7], [12], [20]. Due to the behavior in TLC we concluded that the second compound additionally contained a phosphonyl moiety. We show here that this second compound is 5′-phospho-α-d-ribosyl 1′-phosphonate. In the case of 2AmEtPn-grown cells the accumulated compound is 5′PRib1′2NAcAmEtPn. We previously postulated that 5′-phospho-α-d-ribosyl 1′-phosphonate is the substrate of C–P lyase. This was subsequently shown by *in vitro* analysis to be the case [5]–[7]. Thus, the detection of 5′PRib1′2NAcAmEtPn in the culture medium of *E. coli* is the first demonstration *in vivo* of a substrate for C–P lyase. The fact that 5′PRib1′2NAcAmEtPn carries an acetyl group definitively proves that acetylation of aminoalkylphosphonate precedes C–P bond cleavage by C–P lyase.

The facts that *phnO* strains efficiently utilize 2AmEtPn as P_i_ source and that *phnO^+^* strains efficiently acetylate 2AmEtPn raises the question whether there is simultaneous utilization of 2AmEtPn, acetylated and non-acetylated compounds. Our data indicate that in *phn* wild-type strains there is one dominant pathway. Thus, in the low-field region of ^31^P NMR spectroscopy only a single C–P containing compound was observed (δ 23.6 ppm, Table 3). This chemical shift is consistent with Rib1′N2AcAmEtPn, *i.e.* 2AmEtPn acetylated and attached to ribose. Had there been also a non-acetylated derivative present (*i.e.* α-d-ribosyl 1′-*N*-(2-aminoethylphosphonate)), two peaks had been expected in this region of the ^31^P NMR spectrum. However, the concentration of such an intermediate, signifying a second pathway, may be below the detection limit of ^31^P NMR spectroscopy.

Figure 1 shows the conversion of phosphonate to phosphate ion. Although the structure of most of the intermediates have now been discovered, the terminal steps of the pathway have not firmly been set. It has been suggested that PRPP is the product of the *phn*-specified reactions. PRPP is the product of *phnN* specified ribosyl 1,5-*bis*phosphate phosphokinase activity. The processing of PRPP may involve the activity of one or more phosphoribosyltransferases (PRTases), which produce PP_i_, followed by the activity of inorganic diphosphatase, which completes the formation of P_i_ [6].This pathway is attractive, as organisms such as *E. coli* contain 10 PRTases. PRPP is an important intermediate of purine, pyrimidine and pyridine nucleotide biosynthesis as well as histidine and tryptophan biosynthesis. At least purine and pyrimidine PRTases are generally constitutively synthesized, and, thus, present at all times and available for diphosphorolysis of PRPP formed also by phosphonate degradation [21]. In addition, inorganic diphosphatase, specified by *ppa*, is essential for *E. coli*, and, similarly to PRTases, present at all times [22].

Although we did not perform a detailed kinetic analysis of *E. coli* aminoalkylphosphonate *N*-acetyltransferase, we noticed at least one difference from the *S. enterica* aminoalkylphosphonate *N*-acetyltransferase. The *E. coli* enzyme was able to acetylate R1AmEtPn, which is not a substrate for the *S. enterica* enzyme [9]. Kinetic analysis of the *S. enterica* enzyme revealed that S1AmEtPn was the most efficient substrate (*k*_cat_/*k*_M_ value 7.8×10^4^ M^−1^ s^−1^) compared to 4.1×10^3^ and 5.0×10^3^ M^−1^ s^−1^ for AmMePn and 2AmEtPn, respectively. Additionally, the *k*_cat_/*k*_M_ values diminished for 1-aminoalkylphosphonates with longer alkyl chains [9]. Thus, the high *k*_cat_/*k*_M_ value for S1AmEtPn is consistent with a dual function of acetylation in detoxification and catabolism. Perhaps the acetylation of aminoalkylphosphonates other than AmMePn and S1AmEtPn is without a physiological function, but has evolved as a redundant side effect.

Finally, our results may be applicable to the degradation mechanism of environmental AmMePn, also called AMPA. AMPA/AmMePn is an important metabolite in the catabolism of glyphosate (*N*-(methylphosphono)glycine), the active compound of the herbicide Roundup. Glyphosate can be degraded by either of two pathways both of which involve C–P lyase. In one pathway C–P lyase cleaves glyphosate to P_i_ and *N*-methylglycine (sarcosine), which is further degraded in intermediary metabolism. In the other pathway glyphosate is cleaved to AmMePn and glyoxylate, which is catabolized through the glyoxylate cycle [23]. AmMePn very likely is converted to P_i_ and *N*-methylacetamide by the C–P lyase pathway with the inclusion of the *phnO* gene product as described in Figure 1. In some organisms *N*-methylacetamide may be further degraded as it has been reported that AmMePn is catabolized to CO_2_ [24]. Alternatively, glyphosate-catabolism may include a different acetylation step. Thus, an enzyme with glyphosate *N*-acetylation activity has been discovered in *Bacillus licheniformis*. The physiological importance of this enzyme remains to be elucidated [25].

## Materials and Methods

### General

Organophosphonates, d-alanine, d,l-alanine and acetylcoenzyme A were obtained from Sigma-Aldrich. NMR spectra were recorded on Bruker Avance 400, 500 or 600 MHz spectrometers. ^1^H NMR chemical shifts (δ) are reported relative to HDO, whereas ^31^P NMR chemical shifts are reported relative to 17 mM phosphoric acid as an external standard. ESI-MS analysis was purchased at Queen's University Mass Spectrometry and Proteomics Unit and was performed with an Applied Biosystems/MDS Sciex QStar XL MS instrument.

### Bacterial strains and growth conditions

The *E. coli* K-12 strains used as well as their construction are shown in Table 5. In strain HO2735 the *lacI* ^q^-specified repressor served to repress transcription of genes harbored in the pUHE23-2 vector. *phn*(EcoB) indicates that the *phn* operon originates from phosphonate growth-proficient *E. coli* B. *phn*(EcoK^0^) designates that the *phn* operon originates from wild-type *E. coli* K-12, which is phosphonate growth-deficient, due to an 8-bp duplication in the *phnE* cistron. *E. coli* K-12 strains can be made phosphonate growth-proficient by selection for growth with phosphonate as sole P_i_ source. These phosphonate growth-proficient mutants have lost the 8-bp duplication and are designated *phn*(EcoK^+^) [26], [27]. Liquid growth medium was Tris-buffered 03P minimal medium containing 0.3 mM P_i_, or P_i_-free Mops-buffered minimal medium [12], [28]. Glucose (0.2%) was used as carbon source. Organophosphonates were used at concentrations of 0.3 or 2.0 mM. d,l- and d-alanine were used at a concentration of 100 mg L^−1^. To analyze conversion of phosphonate, cells were grown in 03P medium to an optical density at 600 nm (OD_600_) of 0.45, at which time organophosphonate was added to a concentration of 2 mM. Samples (3 or 5 mL) were removed at time intervals, or alternatively after 20 h of incubation, and centrifuged to remove cells. The supernatant fluid was analyzed immediately or stored at −20°C. Solid medium was Mops-buffered minimal medium and contained 1.8% agar. Glassware and agar were washed with deionized water (Milli-Q system) to reduce undesired P_i_-content.

**Table 5:** *E. coli* strains used.

Strain	Relevant genotype	Reference or construction
BW14001	*Δ(mel-proP-phnCDEFGHIJKLMNOP)2::*Tn*5*seq1/132*(tet)*	[2]
BW14894	*Δ(phnC*?*DEFGHIJKLMNOP)33-30*	[29]
BW17572	*phn*(EcoB) *phnO38::*Tn*phoA'-9*	[6]
BW25113^a^	*phn*(EcoK^0^)	[30]
HO1429	*phn-1*(EcoK^+^)	[7]
HO2536	*phn*(EcoB) *phnJ14::*Tn*phoA'-9 ΔpstS605::cat*	[31]
HO2541	*phn*(EcoB) *phnO38::*Tn*phoA'-9 ΔpstS605::cat*	[6]
HO2542	*phn*(EcoB) *phnP54::*Tn*phnA'-1 ΔpstS605::cat*	[6]
HO2568	*phn*(EcoB) *ΔpstS605::cat*	[31]
HO2678	*Δ(phnC*?*DEFGHIJKLMNOP)33-30*	P1(BW14894)×BW14001 Mel^+^ b
HO2680	*Δ(phnC*?*DEFGHIJKLMNOP)33-30 ΔpstS605*	[31]
HO2735	*Δ(phnC*?*DEFGHIJKLMNOP)33-30*/F *lacI* ^q^ *zzf::*Tn*10*	[6], [32]
HO3412	*ΔpstS605::cat phn*(EcoK^+^) or *phn*(EcoB) *ΔphnO789::kan*	P1(JW4054-4)×HO2568, Kan^rb^
HO3413	*phn*(EcoK^+^) *ΔphnO789::kan*	JW4054-4, MePn as P_i_ source [7]
HO3414	*phn*(EcoK^+^)	BW25113, MePn as P_i_ source [7]
HO3417	*phn*(EcoK^+^) *ΔpstS605::cat*	P1(HO2568)×HO3414, Cml^rb^
HO3418	*phn*(EcoK^+^) or *phn*(EcoB) *phnO38::TnphoA'-9*	P1(BW17572)×HO3414, Kan^rb^
JW4054-4^a^	*phn*(EcoK^0^) *ΔphnO789::kan*	[30]

a Purchased from the Coli Genetic Stock Center, Yale University, New Haven, CT.

b Bacteriophage P1-mediated transduction [33]. Selection was for growth with melibiose as carbon source (Mel^+^), kanamycin resistance (Kan^r^) or chloramphenicol resistance (Cml^r^).

### Purification and characterization by NMR of 2NAcAmEtPn

A culture of strain HO2542 (*phnP*) (250 mL of 03P medium) was grown to an OD_600_ of 0.5 at which time 2AmEtPn was added to 2 mM and incubation continued for 24 h. Following centrifugation the supernatant fluid was loaded on a formate form of an AG1-8X column (2.5×30 cm). After wash with 300 mL of deionized water, a gradient of 0–4.0 M ammonium formate in 0.1 M formic acid was used for elution. The flow rate was 0.5 mL min^−1^. ^31^P NMR was used to analyze fractions for phosphorus-containing compounds. Quantification was achieved by the inclusion of an external standard of 17 mM phosphoric acid. The elution profile is shown in Figure S2A. Fractions showing ^31^P NMR chemical shift 18.4 ppm (*i.e.* elution volume 97 to 115 mL) were pooled and the solvent removed repeatedly *in vacuo* to afford 2NAcAmEtPn.

### Purification and characterization by NMR of 5′PRib1′2NAcAmEtPn

The two fractions representing elution volume 125 to 130 (6 mL) (Figure S2B) were combined, lyophilized repeatedly *in vacuo* to afford 5′PRib1′2NAcAmEtPn. As the elution profile was established by ^31^P NMR of each fraction, the final fraction of 5′PRib1′2NAcAmEtPn contained one or more additional compound(s) without phosphorus atoms as contaminants.

### DNA methodology

A *phnO* variant specifying aminoalkylphosphonate *N-*acetyltransferase with a six-histidine tail at the carboxy terminus (*phnO*_C6xHis_) was prepared by PCR with the four deoxyribonucleoside triphosphates, the oligodeoxyribonucleotides 5′-GA*GAATT*CATTAAAGAGGAGAAATTAACT**ATG**CCTGCTTGTGAGCTTCGCCCGGCCACGC-3′ and 5′-TGT*CCATGG* **TTATTA**atggtgatggtgatggtgCAGCGCCTTGGTGAAGCGGAAGTGGCTCTGCTCGTAGCCTTCGCGC-3′ as primers (nucleotides specifying translation initiation and stop codons are shown in bold and nucleotides specifying a hexahistidine-tail are shown in lower case), DNA of strain HO1429 as the template and Vent DNA polymerase (New England Biolabs). The resulting DNA fragment was restricted by *Eco*RI and *Nco*I (recognition sites are shown in italics in the sequences above) and the liberated 479-bp DNA fragment was ligated to similarly restricted DNA of pUHE23-2 (provided by H. Bujard, University of Heidelberg, Germany). The insert of the resulting plasmid, pHO512, was sequenced and found to have the expected nucleotide sequence including the six histidine-specifying codons.

### Purification and assay of aminoalkylphosphonate *N*-acetyltransferase

To purify *phnO*-specified aminoalkylphosphonate *N*-acetyltransferase, strain HO2735 (*Δphn*)/F *lacI* ^q^ *zzf::*Tn*10*, pHO512 (*phnO*_C6xHis_) was grown in LB (0.3 L) at 37°C with aeration by shaking until an OD_600_ of 0.7 was reached, at which time the culture was cooled in ice for 30 min and 36 mg of isopropyl β-d-1-thiogalactoside was added to induce *phnO* gene expression. Incubation then continued with shaking at 27°C for six hours. Cells were harvested by centrifugation, resuspended in 25 mM sodium phosphate buffer, 0.3 M sodium chloride, 10 mM imidazole, 1 mM EDTA, pH 8.0, and homogenized in an Emulsiflex (model C5, Avestin, Ottawa, ON). Debris was removed by centrifugation, and the supernatant fluid was loaded on a 2-mL column of Ni-NTA agarose (Qiagen, Hilden, Germany). After wash with 25 mM sodium phosphate buffer, 0.3 M sodium chloride, 20 mM imidazole, pH 8.0, protein was eluted with repeated additions of 0.5 mL of 25 mM sodium phosphate buffer, 0.3 M sodium chloride, 0.25 M imidazole, pH 8.0. Elution of aminoalkylphosphonate *N*-acetyltransferase was followed by SDS-PAGE. Fractions 7 to 10 were pooled and dialyzed against 25 mM sodium phosphate buffer, pH 8.0. The purity of aminoalkylphosphonate *N*-acetyltransferase was assessed as more than 95% as evaluated by SDS-PAGE.

To assay the activity of aminoalkylphosphonate *N*-acetyltransferase, purified enzyme (10 µL) was added to a reaction cocktail (final volume of 0.6 mL) with final concentrations of aminoalkylphosphonate, acetylcoenzyme A and magnesium chloride of 1, 3, and 3 mM, respectively and 25 mM Tris/HCl buffer, pH 8.0. The disappearance of aminoalkylphosphonate and the appearance of acetylated product were followed by ^31^P NMR.

## Supporting Information

Figure S1 **Growth response of strain HO2568 (** ***phn^+^ ΔpstS*** **) to S1AmEtPn.** The growth medium was 03P. Squares show growth with d,l-alanine present, circles show growth without d,l-alanine. S1AmEtPn was added at time zero. Growth was followed as described in Materials and Methods.
(TIF)

Figure S2 **Elution by ion-exchange chromatography of phosphorus containing compounds generated by strain HO2542 (** ***phnP*** **) after 24 h of incubation at 37°C in the presence of 2AmEtPn.** The used growth medium was added to the column and elution was analyzed by ^31^P NMR spectroscopy. The relative amounts of the various compounds were estimated with an external standard consisting of 17 mM phosphoric acid. Black squares correspond to phosphonate compounds with a chemical shift of δ 24 ppm (Rib1′2NAcAmEtPn for elution at 50 to 97 mL, 5′PRib1′2NAcAmEtPn for elution at 116 to 138 mL); blue squares, δ 20 ppm (2NAcAmEtPn); purple circles, δ 18.6 ppm (Rib1,2cP); red circles, δ 3.6 ppm (5′-phosphate of 5′PRib1′2NAcAmEtPn); green circles, δ 1.6 ppm (P_i_). (A) Elution profile of all five compounds, (B) blow-up of the profile of the compounds with chemical shifts δ 24 (black squares) and δ 3.6 ppm (red circles).
(TIF)

Figure S3 **Time course of conversion of AmMePn by strain HO2542 (** ***phnP*** **).** Squares, AmMePn (δ 9.2 ppm); diamonds, NAcAmMePn (δ 13.9 ppm); triangles, Rib1′NAcAmMePn (δ 17.4 ppm); circles, Rib1,2cP (δ 18.6 ppm).
(TIF)

Figure S4 **Time course of conversion of AmMePn by strain HO2536 (** ***phnJ*** **).** Squares, AmMePn (δ 9.2 ppm); diamonds, NAcAmMePn (δ 13.9 ppm); triangles, Rib1′NAcAmMePn (δ 17.4 ppm).
(TIF)

Figure S5 **^1^H/^1^H COSY spectrum of 5′PRib1′2NAcAmEtPn.** Protons of 2NAcAmEtPn are labeled in red.
(TIF)

Figure S6 **^1^H/^13^C HMBC spectrum of 5′PRib1′2NAcAmEtPn.** Carbons and protons of 2NAcAmEtPn are labeled in red.
(TIF)
