# Supplementary figures and images for: Catabolism and Detoxification of 1-Aminoalkylphosphonic Acids: N-Acetylation by the phnO Gene Product
Source: PLoS One. 2012 Oct 3;7(10):e46416. doi: 10.1371/journal.pone.0046416 (PMC3463581; doi:10.1371/journal.pone.0046416)

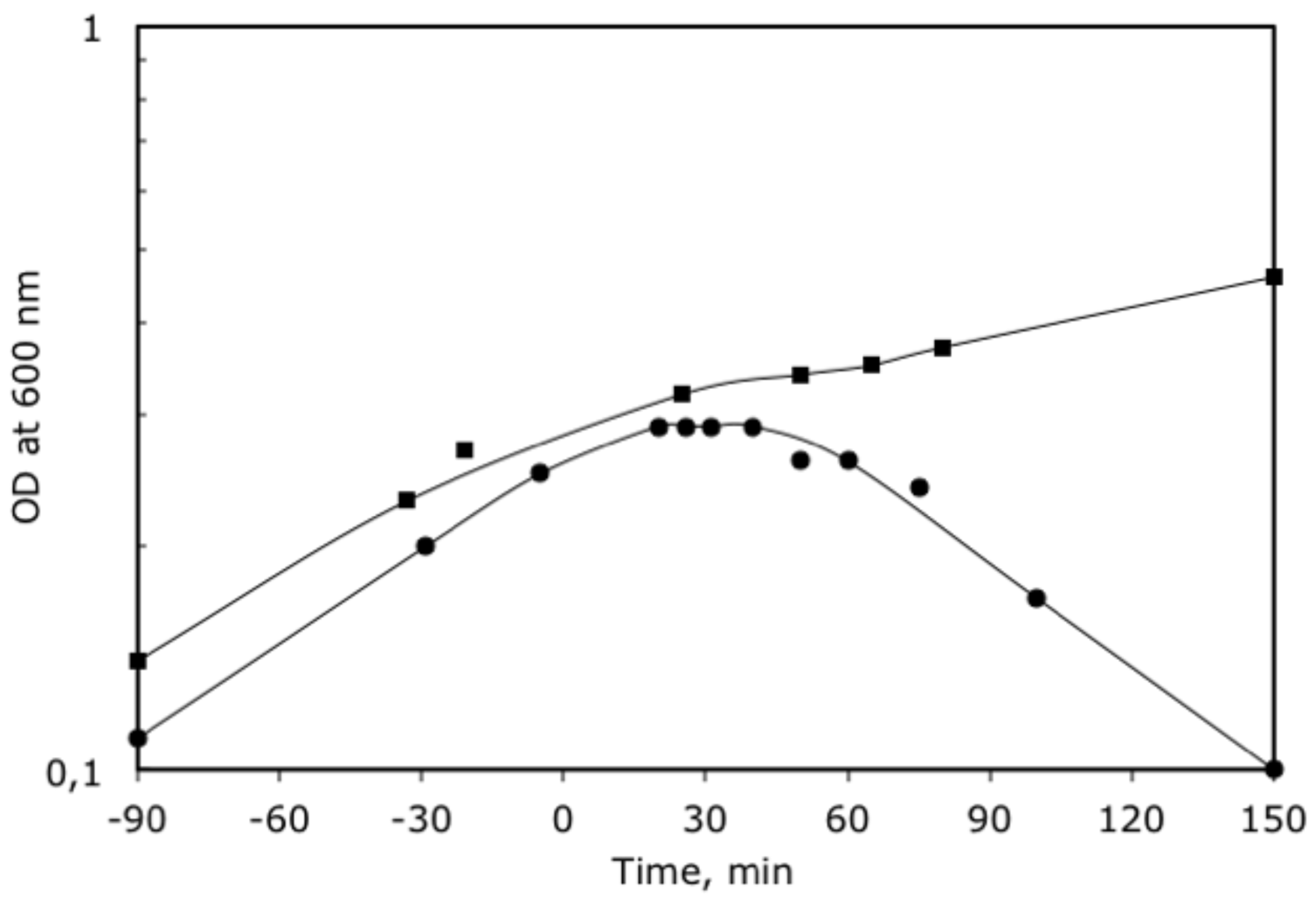

Supplement: Figure S1 — Growth response of strain HO2568 ( phn+ ΔpstS ) to S1AmEtPn. The growth medium was 03P. Squares show growth with d,l-alanine present, circles show growth without d,l-alanine. S1AmEtPn was added at time zero. Growth was followed as described in Materials and Methods. (TIF) [file pone.0046416.s001.tif]

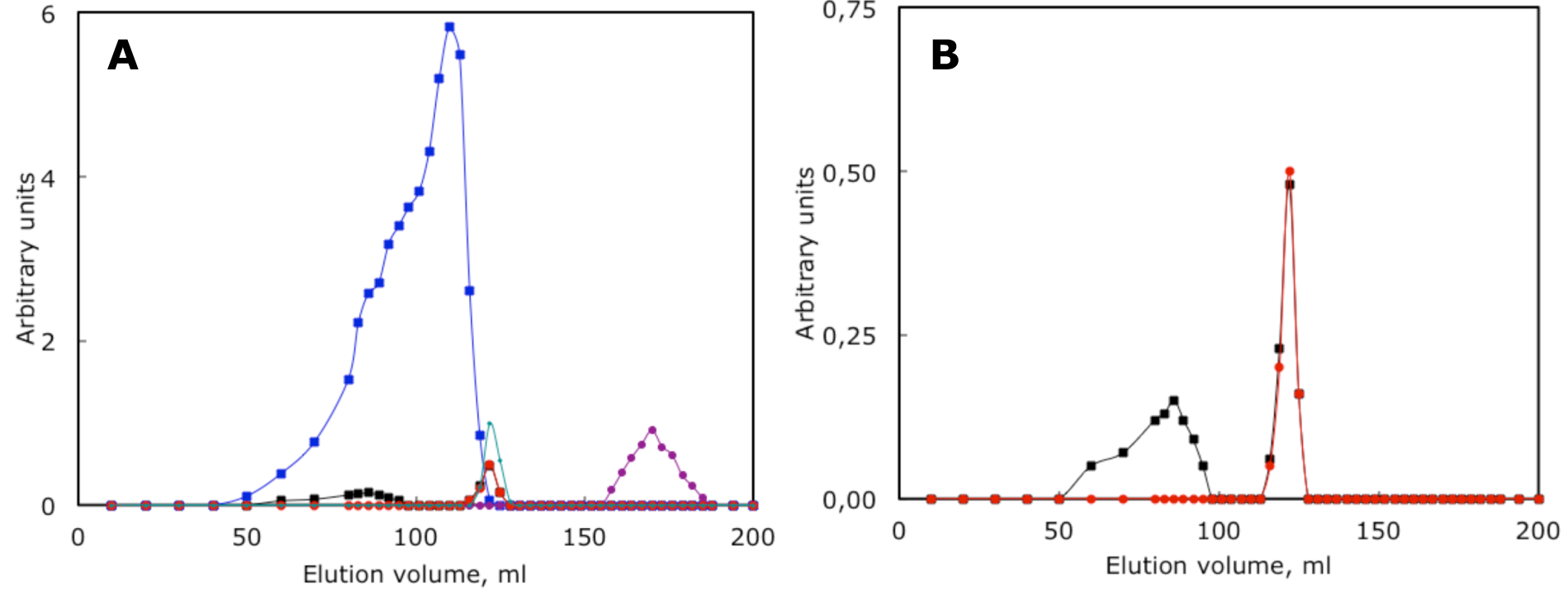

Supplement: Figure S2 — Elution by ion-exchange chromatography of phosphorus containing compounds generated by strain HO2542 ( phnP ) after 24 h of incubation at 37°C in the presence of 2AmEtPn. The used growth medium was added to the column and elution was analyzed by 31P NMR spectroscopy. The relative amounts of the various compounds were estimated with an external standard consisting of 17 mM phosphoric acid. Black squares correspond to phosphonate compounds with a chemical shift of δ 24 ppm (Rib1′2NAcAmEtPn for elution at 50 to 97 mL, 5′PRib1′2NAcAmEtPn for elution at 116 to 138 mL); blue squares, δ 20 ppm (2NAcAmEtPn); purple circles, δ 18.6 ppm (Rib1,2cP); red circles, δ 3.6 ppm (5′-phosphate of 5′PRib1′2NAcAmEtPn); green circles, δ 1.6 ppm (Pi). (A) Elution profile of all five compounds, (B) blow-up of the profile of the compounds with chemical shifts δ 24 (black squares) and δ 3.6 ppm (red circles). (TIF) [file pone.0046416.s002.tif]

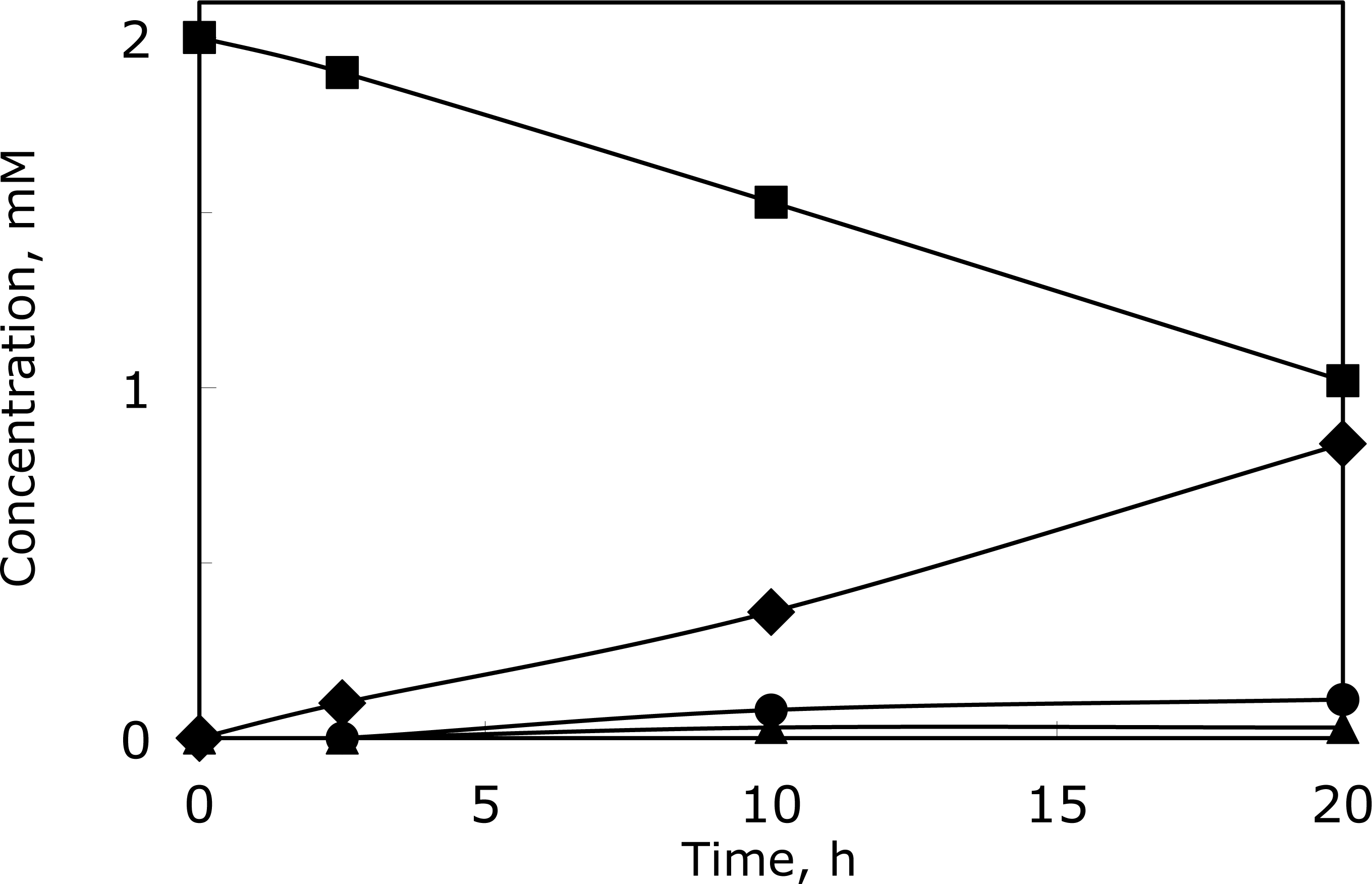

Supplement: Figure S3 — Time course of conversion of AmMePn by strain HO2542 ( phnP ). Squares, AmMePn (δ 9.2 ppm); diamonds, NAcAmMePn (δ 13.9 ppm); triangles, Rib1′NAcAmMePn (δ 17.4 ppm); circles, Rib1,2cP (δ 18.6 ppm). (TIF) [file pone.0046416.s003.tif]

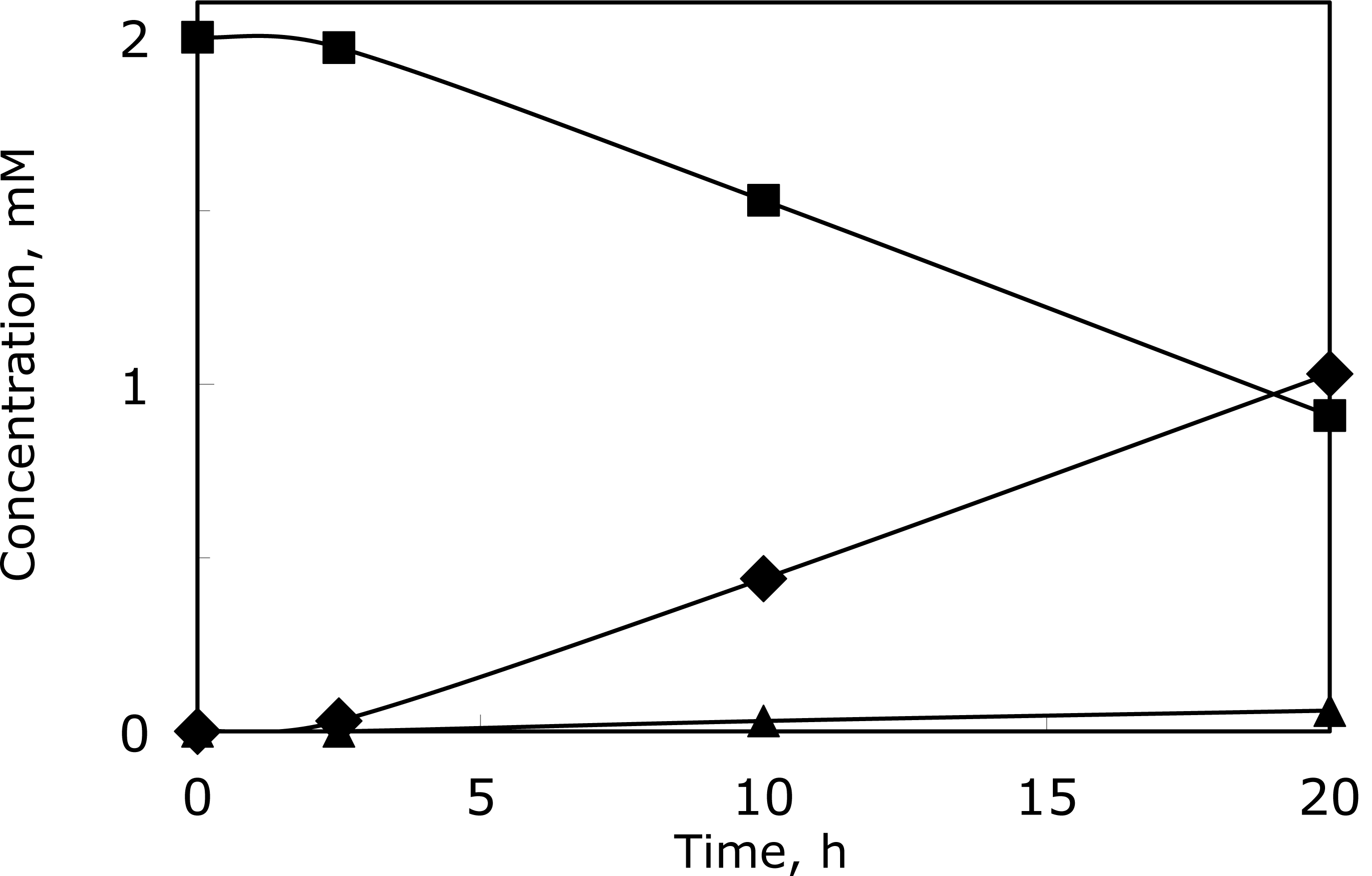

Supplement: Figure S4 — Time course of conversion of AmMePn by strain HO2536 ( phnJ ). Squares, AmMePn (δ 9.2 ppm); diamonds, NAcAmMePn (δ 13.9 ppm); triangles, Rib1′NAcAmMePn (δ 17.4 ppm). (TIF) [file pone.0046416.s004.tif]

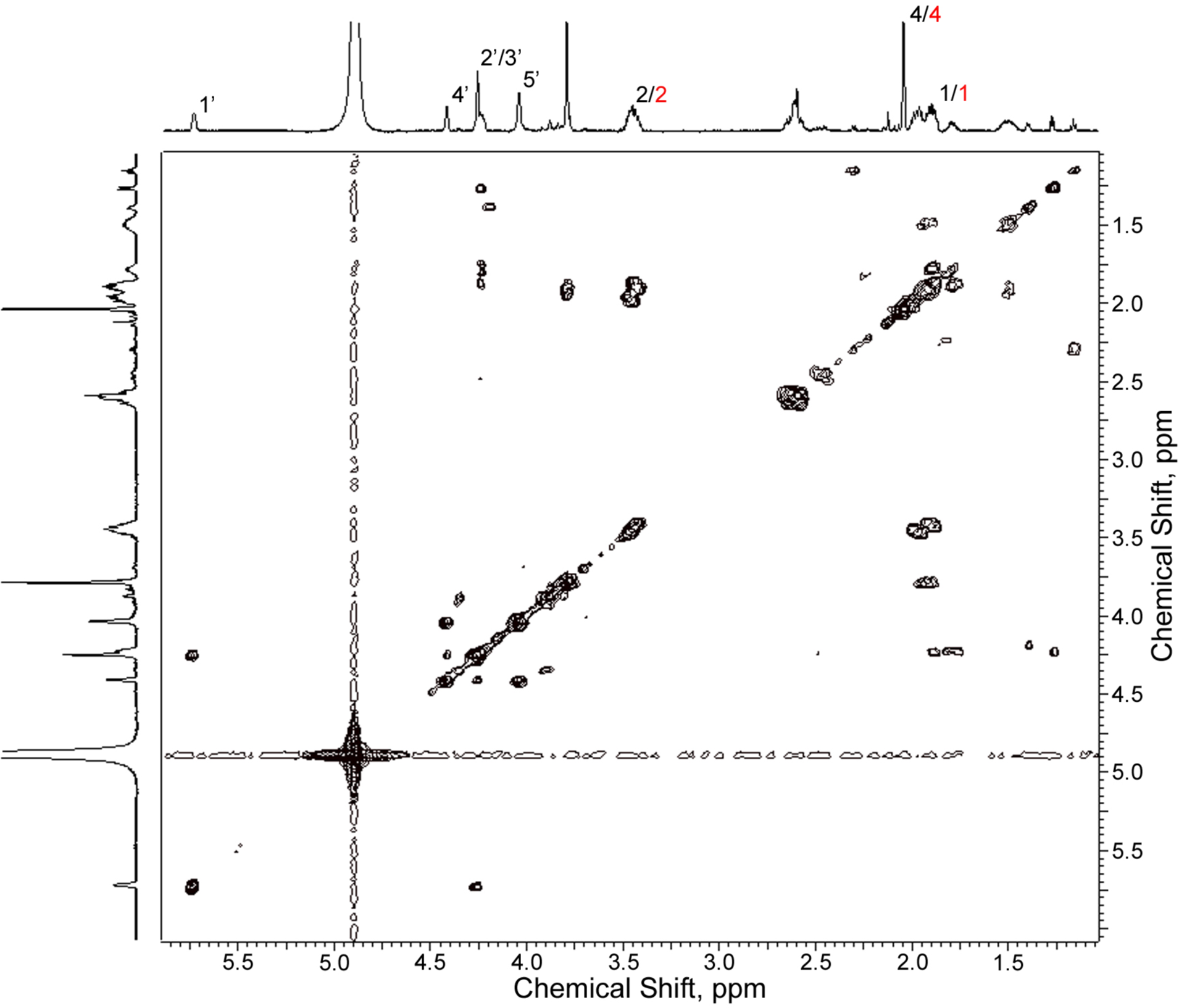

Supplement: Figure S5 — 1H/1H COSY spectrum of 5′PRib1′2NAcAmEtPn. Protons of 2NAcAmEtPn are labeled in red. (TIF) [file pone.0046416.s005.tif]

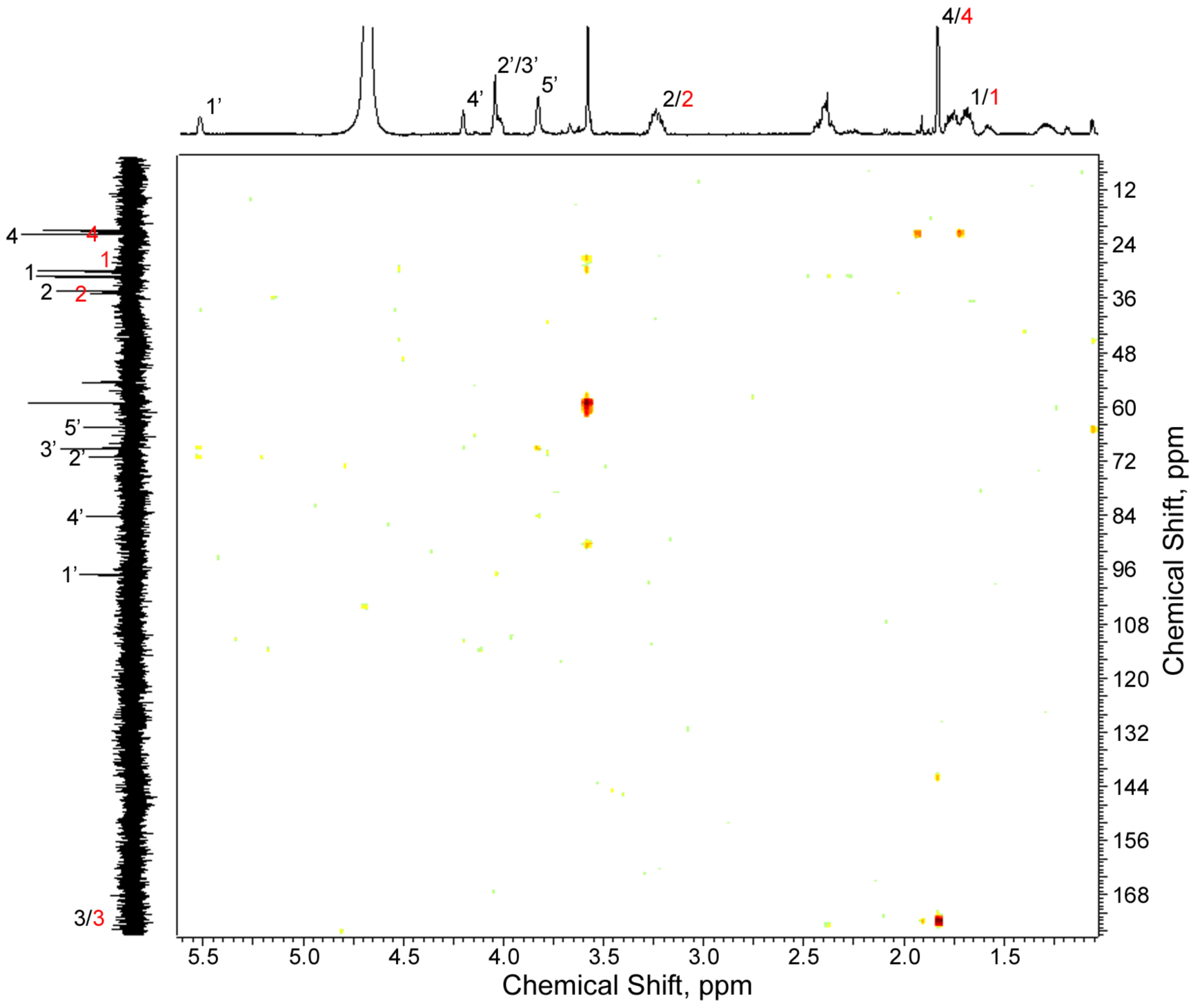

Supplement: Figure S6 — 1H/13C HMBC spectrum of 5′PRib1′2NAcAmEtPn. Carbons and protons of 2NAcAmEtPn are labeled in red. (TIF) [file pone.0046416.s006.tif]
